# Metal-polyphenol-network coated R612F nanoparticles reduce drug resistance in hepatocellular carcinoma by inhibiting stress granules

**DOI:** 10.1038/s41420-024-02161-6

**Published:** 2024-08-28

**Authors:** Yue Zhou, Tongjia Zhang, Shujie Wang, Zitao Jiao, Kejia Lu, Xinyi Liu, Hui Li, Wei Jiang, Xiaowei Zhang

**Affiliations:** 1 Department of Biochemistry and Molecular Biology, School of Basic Medical Sciences, Beijing Key Laboratory of Protein Posttranslational Modifications and Cell Function, Peking University Health Science Center, Beijing, 100191 P. R. China; 2 Shanxi Province Cancer Hospital/Shanxi Hospital Affiliated to Cancer Hospital, Chinese Academy of Medical Sciences, Cancer Hospital Affiliated to Shanxi Medical University, Taiyuan, 030000 P. R. China

**Keywords:** Cancer therapeutic resistance, Tumour biomarkers

## Abstract

Stress granules (SGs) are considered to be the nonmembrane discrete assemblies present in the cytoplasm to cope with various environmental stress. SGs can promote the progression and drug resistance of hepatocellular carcinoma (HCC). Therefore, it is important to explore the mechanism of SG formation to reduce drug resistance in HCC. In this study, we demonstrate that p110α is required for SGs assembly. Mechanistically, the Arg-Gly (RG) motif of p110α is required for SG competence and regulates the recruitment of SG components. The methylation of p110α mediated by protein arginine methyltransferase 1 (PRMT1) interferes with the recruitment of p110α to SG components, thereby inhibiting the promotion of p110α to SGs. On this basis, we generated metal-polyphenol-network-coated R612F nanoparticles (MPN-R612F), which can efficiently enter HCC cells and maintain the hypermethylation state of p110α, thereby inhibiting the assembly of SGs and ultimately reducing the resistance of HCC cells to sorafenib. The combination of MPN-R612F nanoparticles and sorafenib can kill HCC cells more effectively and play a stronger anti-tumor effect. This study provides a new perspective for targeting SGs in the treatment of HCC.

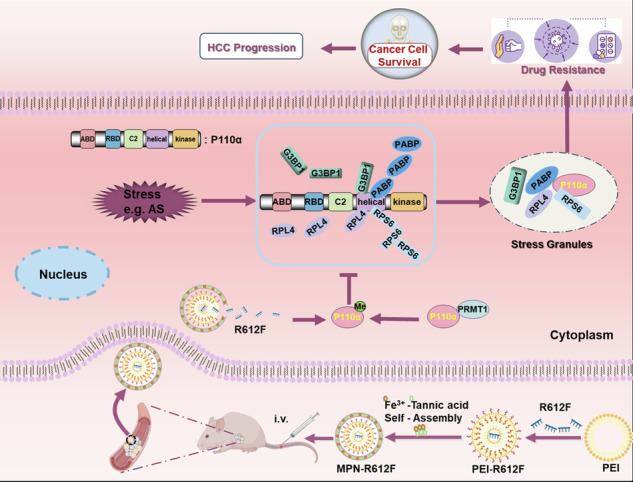

## Introduction

Hepatocellular carcinoma (HCC) has been known as the most common malignant tumor, which has been a long-term threat to human health [1, 2]. The early prognosis of HCC is good, but due to its strong concealment, most of it has entered the middle to late stage when detected [3]. The efficacies of current treatments are insufficient, consequently with a higher mortality rate [4]. Chemotherapy is the best choice for patients who do not meet the conditions for surgical treatment. Sorafenib is a multi-kinase inhibitor that can promote cell apoptosis, reduce angiogenesis, and inhibit tumor cell proliferation, and is currently an effective first-line treatment for advanced HCC. Unfortunately, the development of resistance to sorafenib is becoming more common. Adjibade, et al report that sorafenib is a potent inducer of stress granules (SGs) in cancer cells from various origins, including HCC, prostate cancer, breast cancer, cervical cancer cells [5]. Correspondingly, the formation of SGs contributes to HCC cells resistance to sorafenib.

SGs are considered as membraneless organelles, which are protective mechanisms of cells against external stress. They are mainly composed of stagnant translation preinitiation complexes (PICs), including ribosome sized subunits, translation initiation factors, and many RNA binding proteins [6, 7]. There are many reasons for the formation of SGs, such as oxidative stress, endoplasmic reticulum stress, heat shock, hypoxia, hunger, viral infection, or drug induction [7, 8]. SGs have been confirmed to exist in a variety of cancers, and the high expression of some core proteins of SGs is often positively related to HCC [9, 10]. At the same time, when HCC cells are exposed to environmental stress like chemotherapy, SGs are usually invoked to repair the stress-induced changes and ultimately inhibit HCC cell apoptosis. The continuous accumulation of SGs often leads to an increase in chemotherapy drug resistance [11]. Exploring the mechanism of SGs formation is of great significance for improving HCC therapy strategies.

Class IA phosphoinositide 3-kinases (PI3Ks) belong to lipid kinases that integrate signals from hormones and growth factors to regulate multiple cellular process including proliferation, growth, migration, and cell survival [12, 13]. As a response to the activation of various growth factor receptors, PI3K is recruited onto the cell membrane to phosphorylate phosphatidylinositol (4,5) bisphosphate (PIP2) to generate phosphatidylinositol-3,4,5-triphosphate (PIP3), that is the second messenger required to activate a series of downstream kinases. PI3K consists of catalytic subunits (p110) and regulatory subunits (p85), which have been shown to form a heterodimer. p110α, encoded by *PIK3CA*, an essential isoform of the catalytic subunits, is ubiquitously expressed in various tissues. Somatic mutations of p110α often occur in breast cancer, endometrial cancer, colon cancer, glioblastoma, and prostate cancer [14, 15]. p110α is mainly found in the cytoplasm, but very low levels can be detected in the nucleus of some cells. In addition, p110α was shown to play an important role in cell survival and proliferation. The mutation of *PIK3CA* gene can cause sustained activation of PI3K, leading to uncontrolled cell proliferation and ultimately the formation of tumor cells [16]. Studies have shown that PI3K serves as a driving factor for the formation of SGs, and its pro-stress granules activity may contribute to its carcinogenic ability [17–19], while which isoform of PI3K regulating SGs assembly is still unknown.

Besides, various posttranslational modifications (PTMs) in SGs-nucleating proteins were found to modulate SGs assembly, including phosphorylation, dephosphorylation, poly (ADP) ribosylation, deacetylation, and glycosylation [20]. Recently studies have shown that methylation is an important PTM regulating SG dynamics [21, 22]. Protein methylation at arginine residues is primarily catalyzed by protein arginine methyltransferase (PRMT) family. The PRMT family consists of three types, including nine mammalian PRMT species. Importantly, PRMT1 as the type I PRMTs can catalyze the production of asymmetric dimethylarginine (ADMA) and regulate SG assembly by methylating multiple SG-nucleating proteins, including Ras-GTPase-activating protein SH3 domain-binding protein (G3BP1) and FUS/LTS [23, 24]. It is reported that PRMT1 is an important protection factor from alcohol-induced liver injury and HCC development [25]. These studies suggest that PRMT1-mediated methylation may be involved in HCC progression and resistance to sorafenib through regulation of SGs formation.

Due to the development of cancer therapy methods, nanotechnology has been widely introduced into laboratory research and clinical practice. Among numerous nanomaterials, metal-polyphenol networks (MPNs) have displayed promising potential in biomedical applications since they provide a simple, rapid, and robust way to construct multifunctional nanoassemblies [26–28]. Polyphenol is an organic molecule containing phenolic hydroxyl groups, which is a common compound widely present in natural plants and has been used for anti-inflammatory, antibacterial, and cancer treatment [29–31]. MPN, as a new type of self-assembled nanomaterial composed of metal ions and polyphenols, can be applied for adjuvant cancer treatment due to its biological adhesion, good biocompatibility, multifunctional drug loading, and stimulus responsiveness.

In this study, we demonstrated that p110α recruits SGs components via its Arg–Gly (RG) motif, which is crucial for its ability to promote SGs formation. The RG motif in p110α is asymmetrically dimethylated by PRMT1, and the increase of arginine methylation in p110α inhibits SGs assembly, thereby inhibiting HCC resistance to sorafenib and HCC progression. Based on our findings, we constructed a nanomaterial named MPN-R612F (the arginine at position 612 of p110α was mutated to phenylalanine, a mimic of methylated arginine) by introducing tannic acid, a natural polyphenol extracted from green tea and approved by the Food and Drug Administration (FDA), and Fe^3+^ to form MPN on the surface of a polyethylenimine-R612F plasmid complex (PEI-R612F). MPN-R612F has high efficiency, low toxicity, and lysosomal escape function, which increases the targeting and effectiveness of the drug. Meanwhile, MPN-R612F was internalized by HCC cells and significantly inhibited HCC formation. At the same time, combination therapy with sorafenib significantly enhanced the anti-tumor effect of sorafenib. This strategy may provide an innovative strategy for improving the outcome of traditional cancer therapy.

## Results

### p110α interacts with G3BP1 and is involved in SGs assembly in HCC cells

**Fig. 1:**
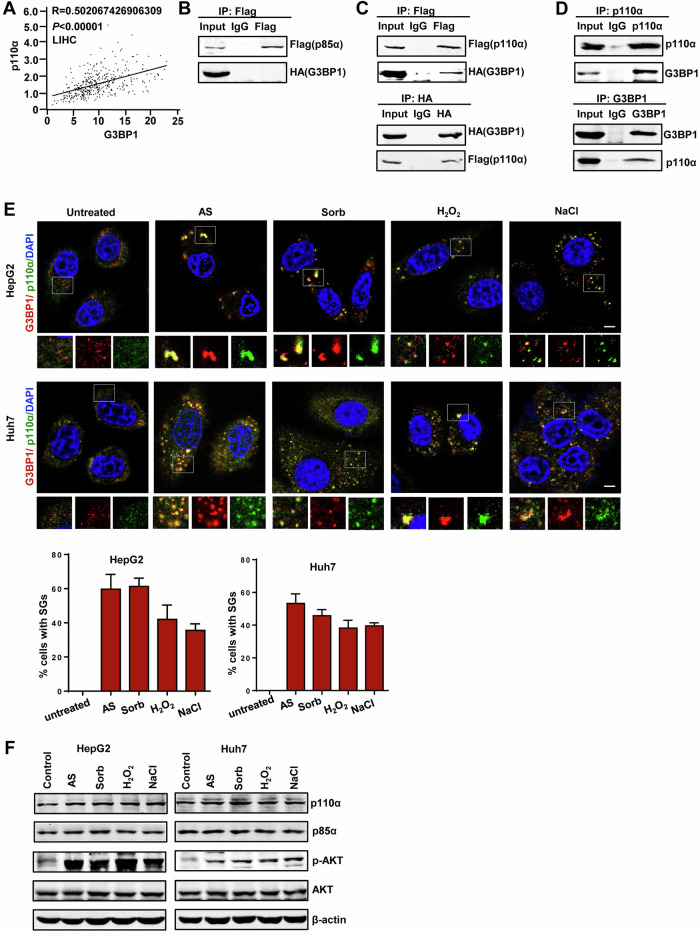
p110α interacts with G3BP1 and is involved in SGs assembly in HCC cells. **A** The correlation analysis of G3BP1 and p110α from TCGA public database. Pearson test was performed. **B** Immunoprecipitation analysis of the exogenous interaction between G3BP1 and p85α in HEK293T cells co-transfected with Flag-p85α and HA-G3BP1. **C** Immunoprecipitation analysis of the exogenous interaction between G3BP1 and p110α in HEK293T cells co-transfected with Flag-p110α and HA-G3BP1, followed by western blotting analysis of HA in the Flag precipitates or of Flag in the HA precipitates. **D** Immunoprecipitation analysis of the endogenous interaction between G3BP1 and p110α in HepG2 cells, followed by western blotting analysis of p110α or of G3BP1 in the precipitates. **E** Double-immunofluorescence analysis of G3BP1/ p110α under various stresses. HepG2 or Huh7 cells were treated with or without 500 μM AS or 1 mM H2O2 for 1 h, or 400 mM sorbitol or 200 mM NaCl for 30 min, and then stained with G3BP1 (red)/p110α (green) as SGs markers and scored. Blue denotes the cell nucleus counterstained by DAPI. **F** Expression of the indicated proteins in various stressed HepG2 and Huh7 cells. The western blotting was performed. β-actin was used as a control. Bars indicate the average percentage of cells with more than three SGs. Data represent the mean ± SD. *n* = 3. Scale bars = 10 μm. LIHC liver hepatocellular carcinoma.

It has been reported that PI3K drives mTORC1 activity to promote stress granule assembly [17], while the specific regulatory mechanism is still unknown. PI3K consists of a catalytic subunit p110 (α, β, or δ) interacting with a regulatory subunit p85 (α or β) [32]. We hypothesized that PI3K might interact with G3BP1, the core protein of SGs. We conducted a study on the public database TCGA and found that p110α and G3BP1 were significantly positively correlated in HCC (Fig. 1A), while other subunits were not significantly correlated with G3BP1 (Fig. S1). Next, we tested whether p110α or p85α interacts with G3BP1 by co-immunoprecipitation (Co-IP) assay. HEK293T cells were co-transfected with the Flag-p110α or Flag-p85α and HA-G3BP1 plasmids. The cells were subjected to IP and western blotting utilizing anti-Flag or anti-HA antibodies. As shown in Figs. 1B and 1C, exogenous p85α did not bind to exogenous G3BP1 (Fig. 1B), whereas exogenous p110α bound to exogenous G3BP1 in vivo (Fig. 1C). Meanwhile, the interaction of endogenous p110α with G3BP1 in HepG2 cells was further detected using Co-IP assay employing anti-p110α or anti-G3BP1 antibody followed by western blotting. Consistent with exogenous results, endogenous p110α bound to endogenous G3BP1 in vivo (Fig. 1D). Among the known proteins in SGs, G3BP1 is considered as the key organizer of SGs assembly, which provides the largest contribution to regulate the properties and composition of SGs [33, 34]. Given that the interaction between p110α and G3BP1, we further investigated the role of p110α in the formation of SGs. HepG2 and Huh7 cells were stimulated with four different SG-inducing chemicals, and then p110α and its binding partner G3BP1 were used as SG markers to determine the role of p110α in SGs under various stress conditions by co-immunofluorescence method. Particles double positive for p110α and G3BP1 were clearly observed in the sodium arsenite (AS), sorbitol (Sorb), H_2_O_2_ and NaCl groups (Fig. 1E), indicating that p110α participates in the formation of SGs induced by various chemicals. Besides, western blotting assays demonstrated that p110α and p85α protein levels remained relatively stable in the presence or absence of the stress stimulus, while phosphorylated AKT protein levels were significantly increased under stress stimulus (Fig. 1F). These data indicated that the catalytic subunit p110α of PI3K is involved in the formation of SGs, and SGs activate the downstream AKT signaling pathway.

### p110α regulates SGs formation in HCC cells

**Fig. 2:**
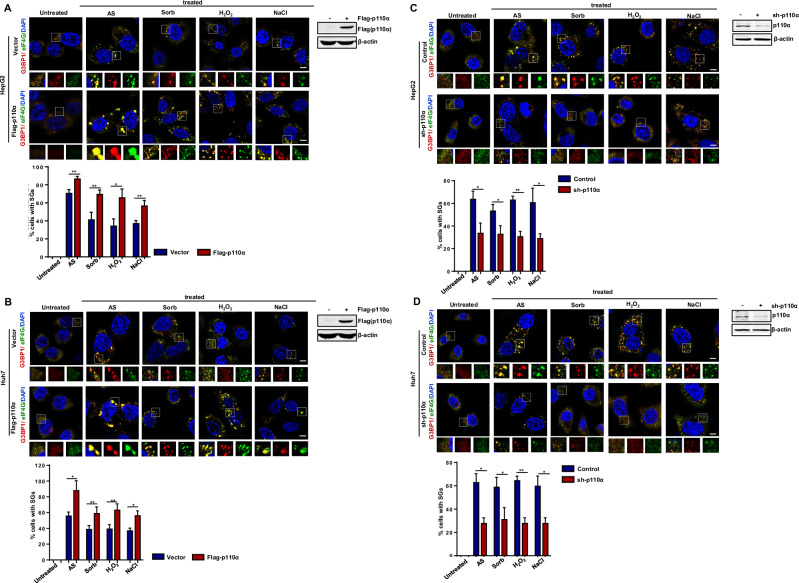
p110α regulates SGs formation in HCC cells. **A**, **B** Left: SGs visualization under various stresses from stresses after p110α overexpression. HepG2 and Huh7 cells were transfected with Flag- p110α, followed by treatment with AS (500 μM, 1 h), sorbitol (400 mM, 30 min), H2O2 (1 mM, 1 h), NaCl (200 mM, 30 min), and then stained for G3BP1 (red)/eIF4G (green) as SG markers. Right: Quantification of SGs. **C**, **D**. Left: SGs visualization under various stresses from stresses after p110α knockdown. Right: Quantification of SGs. HepG2 and Huh7 cells were stably transfected with pLL3.7-p110α through lentiviral infection. Bars denote the average percentage of cells with more than three SGs. Data represent the mean ± SD. *n* = 3. Scale bars = 10 μm. *p < 0.05, **p < 0.01.

We next explored the effect of p110α on the formation of SGs in HepG2 and Huh7 cell lines using a p110α overexpression system and lentivirus-mediated knockdown. Immunofluorescence experiments indicated that p110α overexpression significantly and uniformly promoted the formation of AS-, Sorb-, H_2_O_2_-, and NaCl- induced SGs, as determined by G3BP1 and eIF4G (another typical SG component) as markers (Fig. 2A, B). The immunofluorescence merge images (yellow) denote SGs foci-positive cells. Furthermore, we knocked down p110α expression by shRNA in HepG2 and Huh7 cells and observed its effect on the assembly of SGs induced by four different chemicals. As shown in Fig. 2C, D, SGs detection following p110α knockdown was significantly lower than in those control groups under stress. Moreover, we also detected the formation of SGs after AS removal. As shown in Fig. S2A, p110α overexpression delayed the disassembly of SGs after AS removal. However, SGs were hardly detected in p110α knockdown groups (Fig. S2B). These observations illustrated that p110α not only promotes stress-induced SG assembly, but also delays SG disassembly after stress removal. Taken together, these results revealed that p110α is a core modulator of SG dynamics, responsible for the assembly and disassembly of SGs.

### p110α promotes SGs assembly by enhancing the binding between SGs proteins

**Fig. 3:**
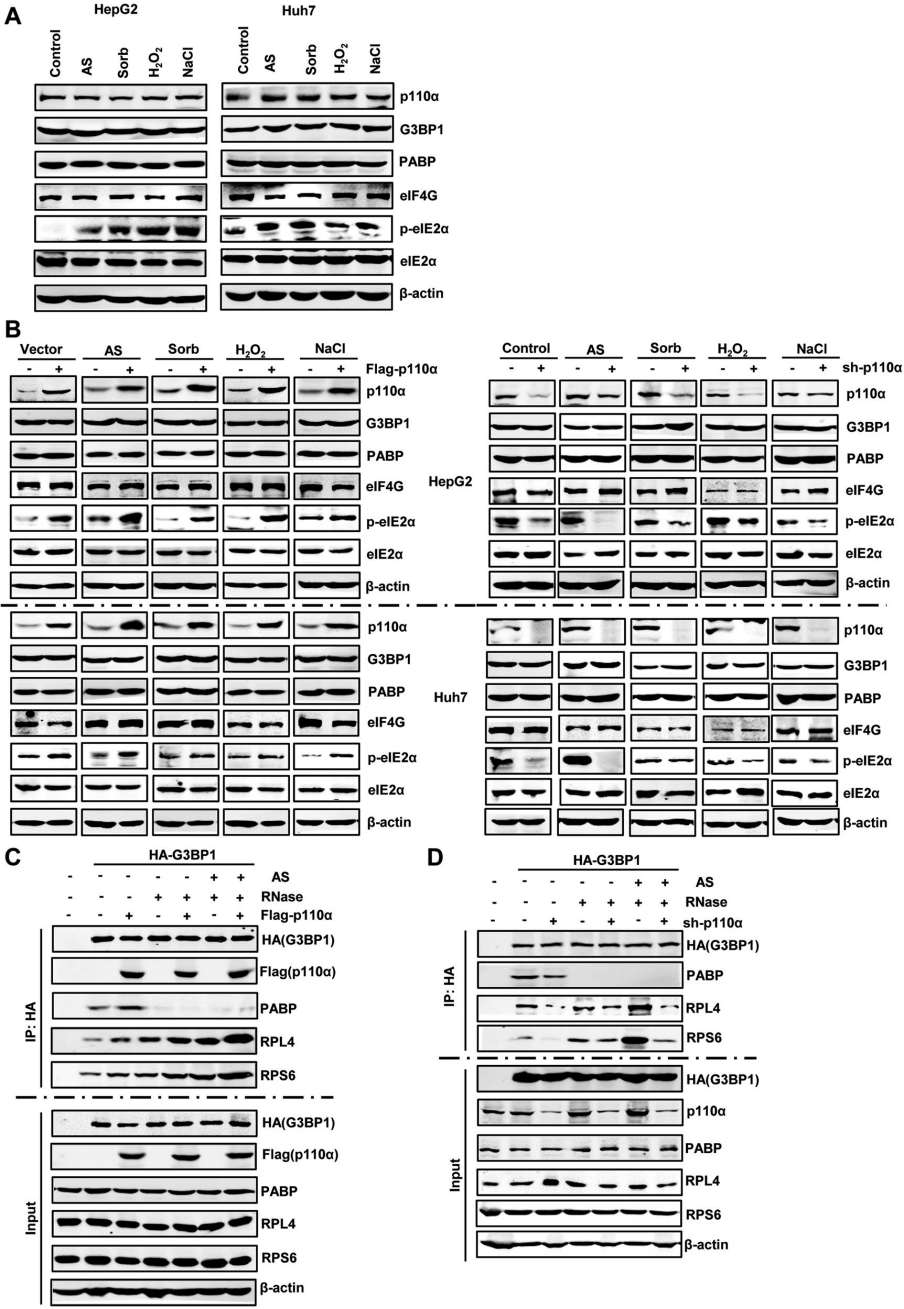
p110α promotes SGs assembly by enhancing the binding between SGs proteins. **A** The expression of relative proteins under stress condition. HepG2 and Huh7 cells were treated with the indicated stresses, and then subjected to western blotting analysis. β-actin was used as a control. **B** The expression of relative proteins in HepG2 and Huh7 cells transfected with Flag-p110α or stably transfected with pLL3.7-p110α under various stresses conditions. β-actin was used as a control. **C** Immunoprecipitation analysis of the associations of G3BP1 with other SGs-nucleating proteins after p110α overexpression. HepG2 cells were transfected with the HA-G3BP1 with or without Flag-p110α, followed by stimulating with or without AS (500 μM, 1 h), in combination with/without RNase A in the lysis buffer. The HA precipitates were analyzed by western blotting to measure the expression of PABP, RPL4 and RPS6. **D** Immunoprecipitation analysis of the associations of G3BP1 with other SGs-nucleating proteins after p110α knockdown.

We investigated the mechanism of p110α-mediated SG assembly. Typically, the phosphorylation of eukaryotic initiation factor 2α (p-eIF2α) leads to the polysome disassembly and subsequent SGs assembly [35]. Therefore, p-eIF2α is considered to be a marker of SG assembly. To assess whether p-eIF2α were activated, western blotting was performed. The results showed that p-eIF2α levels were obviously increased, while p110α protein and other SG protein levels remained relatively stable after four different chemicals treatment (Fig. 3A). These data suggest that stimulation of AS, Sorb, H_2_O_2_ or NaCl can lead to the formation of SGs and that stimulus-mediated regulation of SG dynamics does not involve changes in the expression of p110α itself or other SG proteins. In addition, we found that p110α overexpression or knockdown did not change SG proteins levels, but promoted or inhibited p-eIF2α protein levels (Fig. 3B), confirming that p110α increased SGs assembly without affecting SG protein expression levels. Given that the formation of SGs depends on the interactions between various SG proteins, we tested the possibility of p110α affecting interactions between SG nucleating proteins (G3BP1) and RNA binding proteins (PABP), 60S ribosomal protein (RPL4), or 40S ribosomal protein (RPS6). Co-IP analysis showed that the binding between G3BP1 and PABP, RPL4 and RPS6 was enhanced under AS treatment, and the overexpression of p110α further promoted the binding (Fig. 3C, line 3). The addition of RNase A eliminated the binding between G3BP1 and PABP1, but did not affect the binding between G3BP1 and RPL4 and RPS6 (Fig. 3C, line 4–7). As shown in Fig. 3D, p110α knockdown revealed opposite results compared with p110α overexpression. All together, these data demonstrate that p110α promotes SG assembly by enhancing the binding between SG proteins.

### p110α-mediated SGs assembly depends on its helical domain

**Fig. 4:**
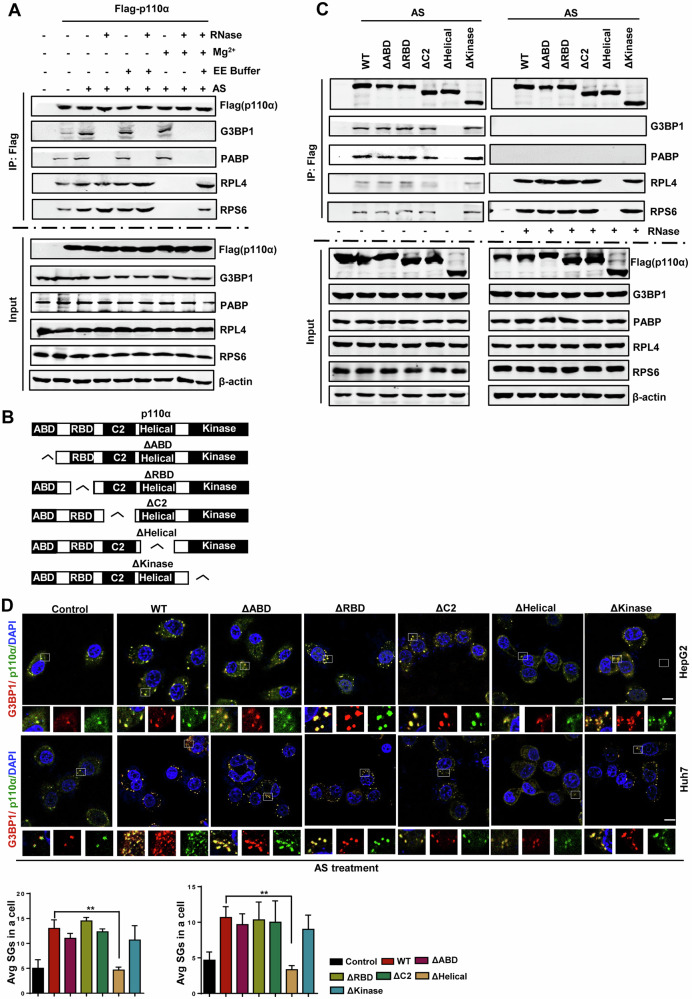
p110α-mediated SGs assembly depends on its helical domain. **A** Immunoprecipitation analysis of the interactions of p110α with SGs-nucleating proteins. HEK293T cells transfected with Flag-p110α were administrated with or without AS, in conjunction with or without RNase A, EE (2 mM EDTA, 2.5 mM EGTA), and/or MgCl2 (5 mM) in the lysis buffer. The Flag precipitates were then subjected to western blotting analysis for the SG proteins. **B** The schematic diagram of a series of mutants. **C** Immunoprecipitation analysis of the interactions of p110α mutants with SGs-nucleating proteins. HEK293T cells transfected with Flag-p110α were administrated with or without AS, in conjunction with or without RNase A, EE (2 mM EDTA, 2.5 mM EGTA), and/or MgCl2 (5 mM) in the lysis buffer. The Flag precipitates were then subjected to western blotting analysis for the SG proteins. **D** Double-immunofluorescence analysis of SGs formation in HepG2 and Huh7 cells expressing Flag-p110α or the indicated mutants, administrating with or without AS (500 μM, 1 h), and then stained with G3BP1 (red)/p110α (green) as SG markers and scored. Bars denote the average number of SGs. Data represent the mean ± SD. *n* = 3. Scale bars = 10 μm. **p < 0.01.

We next investigated the potential interactions of p110α with other SGs-nucleating proteins and ribosomal proteins. Western blotting analysis revealed that p110α bound G3BP1, PABP, RPL4 and RPS6 under normal conditions (Fig. 4A, lane 2), and these interactions increased obviously after AS treatment (Fig. 4A, lane 3). In addition, the binding of p110α to SGs-nucleating proteins G3BP1 and PABP was RNA-dependent, as their binding completely disappeared under RNase conditions (Fig. 4A, lanes 4, 6, 8 and 9), while these interactions were resistant to ribosome-dissociating EDTA-EGTA (EE) or ribosome-stabilizing Mg^2+^ (Fig. 4A, lane 5 and 7). p110α not only bound to the large 60S subunit protein RPL4 but also to the small 40S RPS6 under normal conditions, and their bindings were further enhanced after AS treatment (Fig. 4A, lanes 2 and 3). The binding of p110α to RPL4 and RPS6 was not affected by RNase treatment (Fig. 4A, lane 4), but could be enhanced by EE (Fig. 4A, lane 6), and completely disappeared after Mg2^+^ treatment (Fig. 4A, lane 8) and rescued again by supplementation with EE to neutralize the effect of Mg^2+^ (Fig. 4A, lane 9). These data demonstrate that p110α may directly binding to SGs proteins in EE-dissociated 40S and 60S subunits, which are inaccessible in Mg^2+^-stabilized intact 80S ribosomes, but not to rRNA in subunits, a mechanism vary from that illustrated for G3BP1 [36].

p110α contains five domains, namely ABD (aa 16–105), RBD (aa 187–289), C2 (aa 330–487), helical (aa 517–694) and kinase (aa 765–1051) [37, 38]. To further determine the specific region in p110α required for associations with known SG-nucleating proteins and ribosomal proteins, we constructed a set of deletion mutants (Fig. 4B) and used these mutants in co-immunoprecipitation analysis. The results showed that the deletion of helical domain completely abolished all p110α interactions, whereas deletions of other domains did not, indicating that the helical domain is required for the binding of p110α to SGs-nucleating proteins and ribosomal proteins (Fig. 4C). We also identified the role of each domain in SG condensation in HepG2 and Huh7 cells and found that ΔHelical significantly inhibited SGs formation (Fig. 4D). This confirmed the essential role of the helical domain of p110α in SG assembly. Taken together, these results suggest that the effect of p110α on SGs formation depends on its interactions with SGs nucleating proteins and ribosomal proteins, specifically requiring the helical domain.

### p110α is asymmetrically dimethylated by PRMT1 in HCC cells

**Fig. 5:**
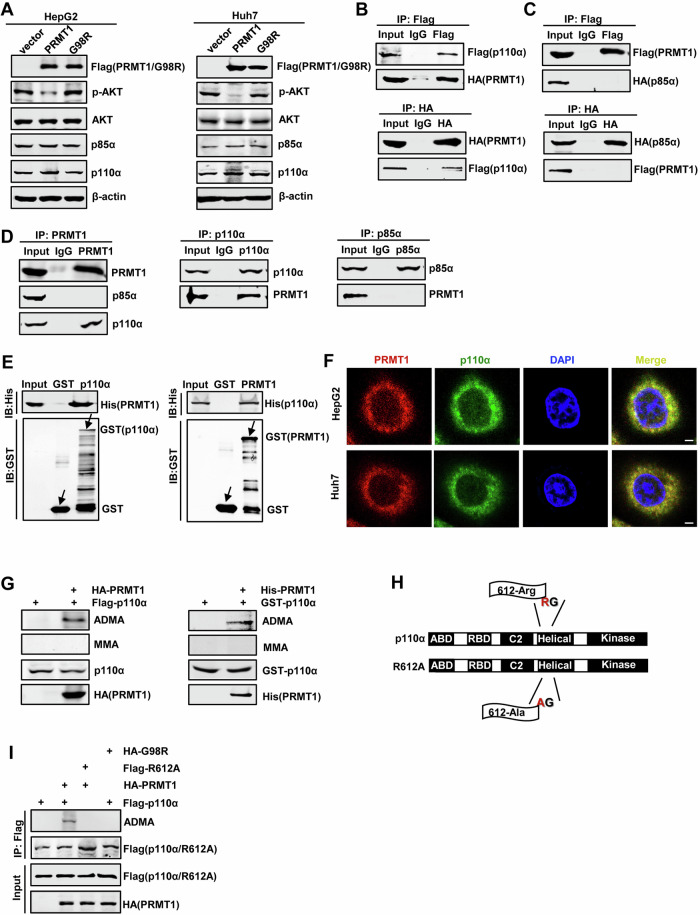
p110α is asymmetrically dimethylated by PRMT1 in HCC cells. **A** The protein levels of the indicated proteins were detected by western blotting in HepG2 and Huh7 cells transfected with Flag-PRMT1 or Flag-G98R. β-actin was used as a control. **B** Immunoprecipitation analysis of the exogenous interaction between PRMT1 and p110α in HEK293T cells co-transfected with Flag-p110α and HA-PRMT1. **C** Immunoprecipitation analysis of the exogenous interaction between PRMT1 and p85α in HEK293T cells co-transfected with Flag-PRMT1 and HA-85α, followed by western blotting analysis of HA in the Flag precipitates or of Flag in the HA precipitates. **D** Immunoprecipitation analysis of the endogenous interaction between PRMT1 and p110α or p85α in HepG2 cells, followed by western blotting analysis of PRMT1 or of p110α or of p85α in the precipitates. **E** GST pull-down analysis of the interaction between PRMT1 and p110α, followed by immunoblotting with anti-His antibody. **F** Immunofluorescence analysis of co-localization of PRMT1 and p110α. Scale bar = 5 μm. **G** The methylation analysis of p110α. **H** Schematic representation of p110α methylation site. **I** Confirmation of p110α methylation mediated by PRMT1. HEK293T cells were co-transfected with Flag-p110α/R612A and HA-PRMT1/G98R and subjected to immunoprecipitation followed by western blotting.

Previous findings have indicated that PRMT1-mediated arginine methylation could be implicated in the SGs formation [39, 40]. To investigate whether PI3K is related to with PRMT1 in vivo, we first performed western blotting analysis on HepG2 and Huh7 cells. As shown in Fig. 5A, compared with the empty vector, PRMT1 overexpression significantly decreased p-AKT levels and did not affect p110α and p85α protein levels. In addition, we constructed the PRMT1 mutant G98R plasmid, which has lost its enzymatic activity, and found that this mutant had no effect on PI3K/AKT signaling pathway, suggesting that PRMT1 might inhibit PI3K/AKT signaling pathway through its enzyme activity. We next sought to investigate whether there is an interaction between PRMT1 and subunits of PI3K. HEK293T cells were co-transfected with HA-PRMT1 and Flag-p110α or HA-p85α and subjected to immunoprecipitation and western blotting with anti-Flag and anti-HA antibodies. The results of reciprocal analysis obviously indicated that exogenous PRMT1 interacts with exogenous p110α rather than p85α in vivo (Fig. 5B, C). We also explored the interactions between the endogenous proteins in HepG2 cells by immunoprecipitation with anti-PRMT1, anti-p110α or anti-p85α antibodies followed by western blotting with indicated antibodies. As shown in Fig. 5D, endogenous PRMT1 bound to endogenous p110α. Furthermore, we verified the binding between PRMT1 and p110α by in vitro GST-PRMT1 or GST-p110α pull-down experiments (Fig. 5E). The interaction of PRMT1/p110α was also detected by immunofluorescence analysis, and the results showed that PRMT1 and p110α were co-localized in the cytoplasm (Fig. 5F). In vitro methylation experiments showed that p110α was asymmetrically dimethylated (ADMA) rather than monomethylated, following addition of PRMT1 (Fig. 5G). It has been reported that PRMT1 can methylate on the RG or RRR motif of the target proteins [41]. In order to confirm the site where p110α is methylated by PRMT1, we analyzed the amino acid sequence of p110α, and found that there was an RG motif (aa 612–613) in the Helical domain, so we mutated the arginine residue at position 612 to alanine (R612A) (Fig. 5H) to test whether p110α could also be methylated by PRMT1. As shown in Fig. 5I, Co-IP experiment confirmed that the R612A mutant could not be methylated by PRMT1. If PRMT1 enzyme activity was absent (G98R), neither wildtype p110α nor mutant R612A could be methylated, indicating that p110α could be specifically methylated by PRMT1, and its methylation site was R612.

### p110α methylation induced by PRMT1 inhibits SGs assembly in HCC cells

**Fig. 6:**
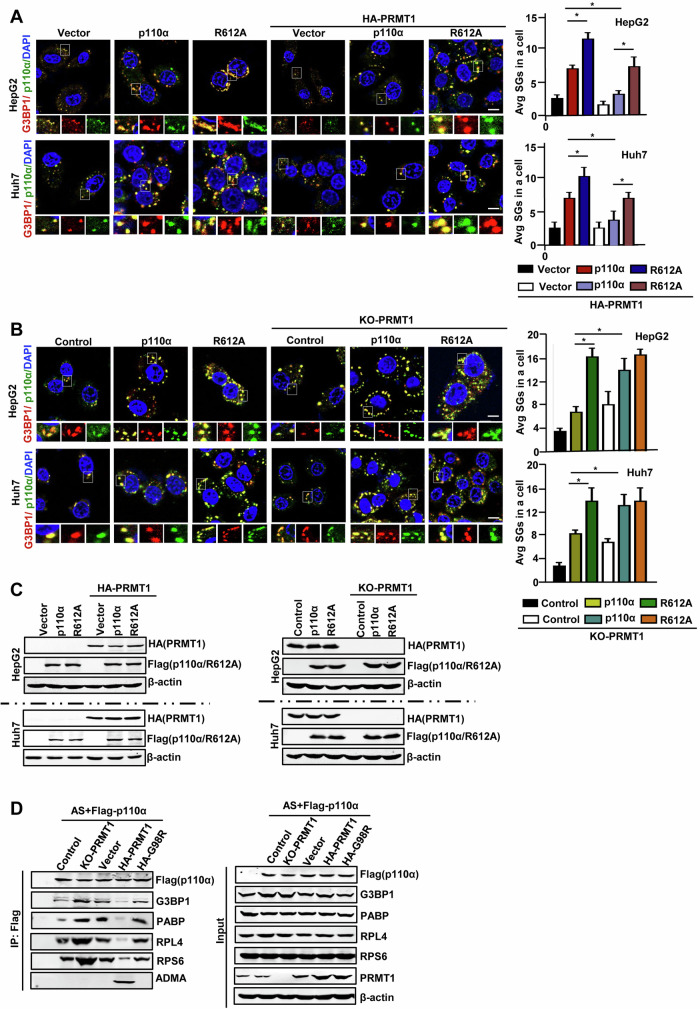
p110α methylation induced by PRMT1 inhibits SGs assembly in HCC cells. **A** Double-immunofluorescence analysis of SGs formation in HepG2 and Huh7 cells expressing Flag-p110α and HA-PRMT1 or Flag-R612A and HA-PRMT1, administrating with AS (500 μM, 1 h), and then stained with G3BP1 (red)/p110α (green) as SGs markers and scored. **B** Double-immunofluorescence analysis of SGs formation in PRMT1 knockout of HepG2 and Huh7 cells expressing Flag-p110α or Flag-R612A, administrating with AS (500 μM, 1 h), and then stained with G3BP1 (red)/p110α (green) as SG markers and scored. **C** Western blot analysis of expression levels of related proteins in (**A**, **B**). **D** Immunoprecipitation analysis of the associations of p110α with SG proteins in PRMT1-KO or HA-PRMT1 or HA-G98R HepG2 cells. Bars denote the average number of SGs. Data represent the mean ± SD. *n* = 3. Scale bars = 10 μm. *p < 0.05. KO knockout.

Since p110α is methylated by PRMT1 and this methylation does not affect the protein level of p110α, does PRMT1-mediated p110α methylation affect SGs assembly in HCC cells? PRMT1 was co-transfected with wild-type p110α or mutant R612A into HepG2 and Huh7 cells, and immunofluorescence assay showed that overexpression of PRMT1 significantly inhibited the role of p110α in promoting SG formation, but had no effect on the role of R612A in promoting SG formation (Fig. 6A). However, PRMT1 knockout significantly enhanced the role of p110α in promoting SG assembly, but had no effect on the role of R612A in promoting SG assembly (Fig. 6B). These results suggest that PRMT1-induced p110α methylation inhibits p110α-mediated SG assembly. Western blotting assays confirmed the aforementioned cell lines were successfully constructed (Fig. 6C). Mechanistically, PRMT1 knockout greatly enhanced p110α interactions with SG nucleators, RPS6 and RPL4, whereas PRMT1 overexpression obviously inhibited the binding of p110α to SGs-nucleating proteins (Fig. 6D). Accordingly, PRMT1 knockout eliminated the p110α ADMA signal (Fig. 6D, lane 3), while PRMT1 overexpression induced the p110α ADMA signal (Fig. 6D, lane 5), suggesting that PRMT1-mediated arginine methylation inhibited the association of p110α with SGs-related elements. Taken together, these results confirmed that PRMT1 is an essential molecular switch that acts a role in the regulation of SG dynamics by mediating p110α arginine methylation.

### The nanoparticle MPN-R612F was designed to enhance the anti-tumor ability of sorafenib

**Fig. 7:**
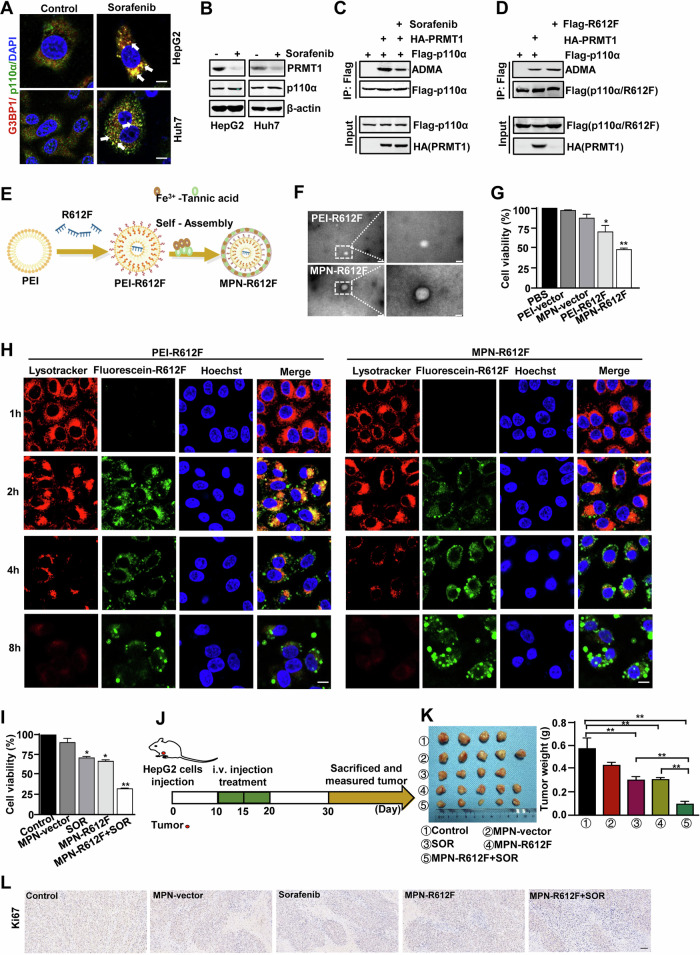
The nanoparticle MPN-R612F was designed to enhance the anti-tumor ability of sorafenib. **A** Double-immunofluorescence analysis of SGs formation in HepG2 and Huh7 cells treated with sorafenib. **B** Western blotting analysis of indicated proteins levels. **C**, **D** Western blotting analysis of ADMA levels. **C** Treatment with sorafenib. **D** Transfection of R612F plasmid. **E** Schematic presentation for the preparation of MPN-R612F. **F** Transmission electron microscopy images of PEI-R612F and MPN-R612F. Scale bar = 100 μm (left)/50 μm (right). **G** Cell viability analysis of HepG2 cells after treated with PBS, PEI-vector, MPN-vector, PEI-R612F, MPN-R612F incubating for 24 h. **H** Lysosome escape analysis of HepG2 cells treated with PEI-R612F or MPN-R612F at 1–8 h by confocal laser scanning microscope. Scale bar = 10 μm. **I** Cell viability analysis of HepG2 cells after treated with MPN-vector, sorafenib, MPN-R612F, or MPN-R612F + SOR incubating for 24 h. **J** Schematic illustration of the animal experimental design model. HepG2 cells were subcutaneously injected into female Balb/c nude mice. After the tumor volume reached to 50 mm^3^, mice were randomized divided into different groups, and then were IV injected with 100 μL of PBS, MPN-vector, MPN-R612F, sorafenib, and MPN-R612F+sorafenib at 10th, 15th, and 20th days. Sorafenib was given at a dose of 5 mg/kg, and DNA in MPN-vector and MPN-R612F was given at a dose of 0.5 mg/kg (*n* = 5 for all groups) in HepG2-tumor-bearing mice. **K** The images of tumor from the indicated groups. Tumor weight was calculated after 30 days. **L** Ki67 expression in indicated groups. Scale bars = 100 μm. Data represent the mean ± SD. *n* = 3. *p < 0.05, **p < 0.01. SOR sorafenib.

Sorafenib is the first first-line drug approved by FDA to treat advanced HCC [42]. The previous study has showed that SGs assembly contributes to HCC resistance to sorafenib [43]. In this study, we also found that sorafenib treatment significantly induced the aggregation of stress granules, while the control group did not (Figs. 7A, S3A). Interestingly, we found that after treatment with sorafenib, PRMT1 protein levels were reduced, while p110α remained unchanged in HepG2 and Huh7 cells (Fig. 7B). Meanwhile, sorafenib treatment significantly reduced the p110α methylation levels (Fig. 7C). These results indicated a certain correlation between sorafenib, SGs, and methylated p110α. Based on our findings that p110α arginine methylation inhibits the formation of stress granules in HCC cells, we wondered whether sustained methylation of p110α could reduce sorafenib resistance in HCC cells by inhibiting the formation of stress granules, thereby killing HCC cells? We constructed a positive (Arg/Phe) mutation, R612F, that simulates constitutive arginine methylation by replacing arginine with phenylalanine, which has similar sized hydrocarbon chains and carries a large hydrophobic moiety, according to the previous report [44]. The hydrophobicity induced by Phe mutation is specific to the methylatable arginine residues, and can simulate the methylation of arginine, causing p110α to exhibit high methylation. Western blotting analysis confirmed that R612F could indeed undergo methylation in the absence of PRMT1, and its methylation effect was similar to that of PRMT1 on p110α (Fig. 7D). Therefore, we planned to design a nanoparticle based on the mutant R612F.

Iron-based nanomaterials, such as iron nanometal glasses, superparamagnetic iron oxide nanoparticles and iron-based metal organic frameworks, have been widely used as anticancer agents [45, 46]. However, the use of toxic materials and cumbersome synthesis routes greatly limit their potential values in biomedical applications. In recent years, MPNs have drawn much attention due to their advantages of easy “one pot” synthesis, high biocompatibility, low cost, and broad application prospects [28]. Inspired by these findings, we combined tannic acid, a polyphenolic compound extracted from green tea, with ferric ions to form MPN on the surface of the polyethylenimine-R612F plasmid complex (PEI-R612F) to generate MPN-R612F (Fig. 7E). When nanoparticles enter the body through the circulation of blood, they selectively accumulate in tumor tissue through permeation and retention effects and then reach the corresponding tumor cells through endocytosis [47]. Under low pH conditions, most of the phenolic hydroxyl groups on tannic acid are protonated, resulting in competitive chelation between proton hydrogen and ferric ions, leading to instability or even disintegration of MPN [28]. Based on these principles, MPN-R612F enter Huh7 and HepG2 cells through endocytosis, ultimately releasing R612F. In detail, the R612F plasmid recombined with PEI through electrostatic interactions to form PEI-R612F complex. The diameter of the PEI-R612F complex obtained by TEM analysis was about 40 nm (Fig. 7F, top panel). Added ferric ions and tannic acid sequentially into the PEI-R612F solution to prepare MPN-R612F, with a diameter increase of approximately 65 nm (Fig. 7F, bottom panel). Previous studies have reported that MPN membranes are a supramolecular network form based on Fe^3+^ and the polyphenol coordination [28]. To demonstrate this, the driving forces for the formation of MPN-R612F were further determined by adding ethylenediaminetetraacetic acid (EDTA), urea, NaCl, and Tween 20 solutions to MPN-R612F, respectively. After EDTA was mixed with MPN-R612F, the color of the solution was immediately bleached. However, urea which breaks hydrogen bonds, NaCl, that can abolish electrostatic forces, and Tween 20, which eliminates hydrophobic forces, were ineffective (Fig. S3B). These observations showed that coordination bonds are the main interaction during the formation of MPN coatings. To investigate the effect of MPN-R612F on cancer cell activity, HepG2 cells were treated with MPN-R612F for 24 h. MTT assay showed that MPN-R612F resulted in the most significant reduction in cell viability, although PEI-R612F treatment also resulted in a slight reduction in cell viability (Fig. 7G). The data suggest that MPN-R612F has better killing ability to cancer cells than PEI-R612F.

Internalized nanoparticles are transported to lysosomes, and once intercepted, they are rapidly dissolved. Therefore, avoiding the degradation of nanoparticles by lysosomes is a prerequisite for successful transfection. To investigate whether MPN-R612F has higher transfection efficiency than PEI-R612F, we performed lysosomal escape assay. As shown in Fig. 7H, lysosomes, fluorescein-R612F, and the nuclei of transfected HepG2 cells were stained at different time points after transfection. Specifically, 1 h after transfection, few fluorescein-R612F entered PEI-R612F-treated cells and MPN-R612F-treated cells. At 2 h, a small amount of fluorescein-R612F entered MPN-R612F-treated cells, while a large amount of fluorescein-R612F was captured in the lysosomes of PEI-R612F-treated cells. Then, at 4 h, some fluorescein-R612F was still entrapped in lysosome of PEI-R612F-treated cells, and there was less fluorescein-R612F than that at 2 h, while more fluorescein-R612F had escaped from lysosomes of MPN-R612F-treated cells. Finally, at 8 h, a large number of fluorescein-R612F escaped from lysosomes and some even located in the nucleus of MPN-R612F-treated cells. However, although fluorescein-R612F also escaped from lysosomes, the intensity of fluorescein significantly decreased in PEI-R612F-treated cells, suggesting that a large number of R612F DNA was lysosomal degradation. These results suggest that MPN-R612F had stronger lysosomal escape ability than PEI-R612F and could function more effectively in cancer cells. We also investigated whether MPN-R612F treatment could inhibit the SGs assembly. The results showed that both MPN-R612F and PEI-R612F could inhibit the formation of SGs, and the inhibition effect of MPN-R612F on SGs was more significant than that of PEI-R612F (Fig. S3C). These observations illustrated that MPN-R612F had potential anti-cancer ability.

Considering the ability of MPN-R612F to inhibit SGs formation and the positive association between sorafenib resistance and SGs, we treated HepG2 cells with the combination of MPN-R612F and sorafenib and to observe whether MPN-R12F could improve the anti-tumor effect of sorafenib. MTT assay showed a greater reduction in cell viability after 24 h of treatment with MPN-R612F and sorafenib compared to treatment with MPN-R612F or sorafenib alone (Fig. 7I). Next, we explored the potential efficiency of our nanoparticles to treat tumors in vivo, and the treatment protocol is shown in Fig. 7J. HepG2 cells were subcutaneously injected into female BALB/c nude mice. When the tumor volume increased to 50 mm^3^, MPN-vector, MPN-R612F, sorafenib, and MPN-R612F with sorafenib were injected into the tail vein on days 10, 15, and 20, respectively. As shown in Figs. 7K and S3D, MPN-R612F combined with sorafenib displayed the most evident inhibitory effect on tumor proliferation compared with other groups. Besides, consistent with the results from cell-derived xenograft, immunohistochemical staining showed a lower rate of Ki67 positive cells in MPN-R612F combined with sorafenib than in MPN-R612F or sorafenib alone (Figs. 7L and S3E). We also observed the effects of MPN-R612F on the liver and kidneys of mice, and found that the nanoparticles did not cause damage to the liver and kidneys of mice (Fig. S3F–I).

In summary, our MPN-R612F nanoparticles could effectively kill tumors by inhibiting the formation of stress granules, and had little damage to liver and kidney. Combined use with sorafenib could significantly enhance the anti-tumor effect of sorafenib.

## Discussion

The microenvironment of hepatocellular carcinoma is full of stresses, like hypoglycemia, high concentrations of ROS and hypoxia, which trigger SGs formation that can strongly influence the prognosis of patients [48, 49]. SGs not only adapts tumor cells to the microenvironment, but also modulates tumor metabolism and the expression of oncogenes, which is directly corelated to tumors development and anticancer drugs efficiency [50]. Therefore, targeting stress granules would be novel therapeutic strategy for HCC. SGs are a protective mechanism of the cell itself and are products of cells response to external pressure. The formation of SGs is related to many diseases, but so far, SGs have only been detected in a few diseases [51–54] that (1) neurodegenerative diseases such as fragile X-chromosome syndrome, Alzheimer’s disease, amyotrophic lateral sclerosis (ALS), and spinocerebellar ataxia. (2) virus infection. (3) cancers. Research has shown that there is a large amount of SGs aggregation in the brain tissue of Alzheimer’s disease mouse models, and SGs assembly is positively correlated with the disease [55]. In addition, impaired RNA in SGs accumulates in neurons, leading to further exacerbation of the disease [56]. SGs also exist in virus infected cells and can be considered as a host defense response against virus invasion, effectively limiting virus invasion. Inhibiting the formation of SGs increase virus replication and spread [57]. The anti-apoptotic phenomenon in the treatment of tumors is related to the formation of SGs. Research has found that SGs antagonize tumor cell apoptosis by inhibiting the mTOR1 pathway [58]. Recently, PI3K has been confirmed to promote the SGs assembly, but the regulation of which subunit has not been clarified yet.

In this study, we explored the cellular role of the catalytic subunit of PI3K and p110α in regulating SG assembly, and investigated potential molecular mechanisms. We first identified a significant positive correlation between p110α and G3BP1, a known SGs-nucleating protein that determines the SGs formation. Next, we treated cells with different chemicals to stimulate the formation of SGs, and found that p110α is involved in SGs assembly. In detail, p110α overexpression enhanced, while p110α knockdown inhibited, stress granules. Based on this, we further investigated the molecular mechanism of p110α mediating SGs formation. Immunoprecipitation analysis indicated that p110α interacts with SG nucleators and ribosomal proteins, whereas has no effect on levels of these proteins. In addition, the association between p110α and G3BP1 or PABP is RNA dependent, and p110α can directly bind to RPL4 and RPS6 in the 60S and 40S subunits dissociated from EE buffer, which are inaccessible in Mg^2+^ stable intact 80S ribosomes, but not in contact with rRNA in the subunits. Meanwhile, we constructed mutants of p110α and found that the helical domain of p110α determines the role of p110α on the formation of SGs.

Another important discovery of this study is that the methylation condition of the RG motif in p110α is finely regulated by PRMT1 in response to cellular stress. Mechanistically, PRMT1 interacts with p110α, but not with the regulatory subunit, p85α, and p110α can be methylated at R612 by PRMT1. PRMT1 overexpression inhibits, while PRMT1 knockout promotes, p110α interaction with SG nucleating-proteins, RPL4 and RPS6, through which the arginine methylation of p110α modulates SG dynamics. These observations support the previous view that arginine methylation and RG motif are associated with protein-protein interactions, nucleic acid binding, and signal transduction, except for nuclear/cytoplasmic shuttling [59, 60]. We also found that when Arg-612, the site of p110α with ADMA modification, is mutated to Ala, the methylation state p110α completely disappears and the effect of p110α on SGs formation significantly is rescued. Therefore, we believe that targeting the arginine methylation of p110α is the promising therapeutic strategy for treating diseases that worsen due to increased SGs.

According to our findings about PRMT1/ p110α to regulate SGs formation, we first generated the mutant of p110α, R612F, a plasmid replacing arginine with phenylalanine that can mimic constitutive arginine methylation. Ferric ions and tannic acid were used to generate MPN, which was then mixed with the PEI-R612F plasmid to form an MPN-R612F sphere. We discovered that MPN-R612F significantly inhibits hepatoma cells growth and has the stronger lysosomal escape ability than PEI-R612F. Moreover, MPN-R612F obviously inhibits SGs assembly compared with PEI-R612F. Interestingly, the literature reports that PERK-eIF2α-SGs pathway plays an essential role on hepatocarcinoma cells resistance to sorafenib [43]. Inspired this research, we further investigated whether the combination of MPN-R612F and sorafenib can improve the anti-tumor effect of sorafenib. The results demonstrated that the combination of MPN-R612F and sorafenib not only suppresses the proliferation of HCC cells, but also reduces tumor growth in tumor-bearing mice.

**Fig. 8:**
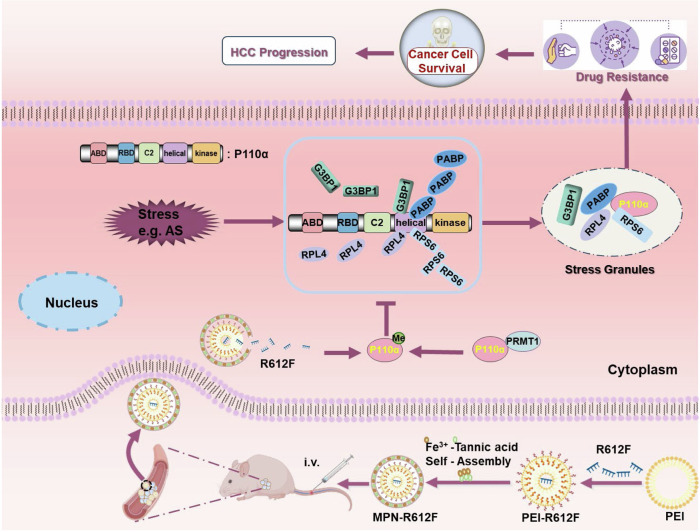
Schematic description for anticancer therapy on methylation of p110α mediated by PRMT1 to inhibit SGs assembly. Various stresses trigger SGs formation and p110α is involved in SGs assembly, interacting with SG nucleators and ribosomal proteins, thereby jointly promoting the formation of SGs. p110α can be methylated by PRMT1 and the methylation of p110α in turn inhibits SGs formation. The PEI-R612F combines with ferric ions and tannic acid to generate MPN-R612F. After MPN-R612F nanoparticles reach the tumor site via passive targeting, R612F inhibits SGs assembly and ultimately prevents cancer cells survival and inhibits HCC progression.

Together, our present study reveals p110α promotes the formation of SGs by regulating the interactions among SGs proteins. Moreover, PRMT1-mediated methylation of p110α acts an important role in the resistance of HCC cells to sorafenib. MPN-R612F combined with sorafenib may overcome the drug tolerance that occurs with conventional anticancer agents and provide a new clinical perspective into HCC therapy where traditional therapeutic strategies have failed. Our results are summarized in Fig. 8.

Cells are inevitably exposed to various adverse conditions, such as oxidative stress, osmotic stress, heat shock, and drug therapy. The induction of SGs is an important and conservative cellular strategy that regulates protein translation and cellular signaling, thereby minimizing stress-related damage and promoting cell survival [61]. After the removal of stress conditions, the SGs exist for 60–120 min, and then spontaneously disassemble. SGs disassembly occur through multiple steps, wherein the stalled RNAs are titrated out of SGs, thereby causing structural instability of the protein complexes, and subsequently stepwise disassembly of the visible SGs. SGs are dynamic, and when the environmental stress disappears, the proteins and RNAs of SGs will undergo “decompression” and return to their original positions to help cells recovery normal operation. Timely disassembly of SGs is essential for maintaining cell viability and protein homeostasis [62].

Especially, tumor cells are exposed to various environmental stress in the tumor microenvironment during tumorigenesis, and SGs are typically used to repair stress-induced changes and ultimately contribute to the characteristics of cancer [63]. Therefore, the sustained accumulation of SGs often correlate with cancer. This study reveals the mechanisms of SGs in tumors and suggests novel directions for HCC treatment. Additionally, this research provides a new insight for the possible application of metal polyphenol coordination network (MPN) targeting SGs in the treatment of HCC. However, this study only suggests the regulation of SGs on drug resistance. It remains to be explored whether SGs function as protective reservoirs for proteins, enabling rapid adaptation to the demands of the tumor microenvironment of HCC. In the future, we will focus on the impact of SGs on the tumor microenvironment of HCC and comprehensively evaluate the role of SGs in the progression of HCC.

## Subjects and methods

### Plasmids

The coding region of p110α was cloned into the p3xFlag-CMV-10 vector which preserved as the seed for the generation of Flag-labeled p110α mutants within the vector including the following deletants: p110α with deleted amino acids 46–315 (Δ46–315, lacking the ABD domain), Δ559–867 (lacking the RBD domain), Δ988–1461 (lacking the C2 domain), Δ1549–2082 (lacking the Helical domain), and Δ2293–3153 (lacking the Kinase domain); and the following point mutant: p110α with the Arg residue in the RG domain mutated to Ala (p110α-R612A). The primers used were listed in the Supplementary Table.

### Cell culture, transfection, lentivirus infection and CRISPR/Cas9

HEK293T, Huh7, and HepG2 cells were obtained from ATCC and were cultured in the Dulbecco’s Modified Eagle Medium (DMEM) supplemented with 10% fetal bovine serum (FBS), 100 μg/mL penicillin, and streptomycin at 37˚C under 5% CO_2_ circumstance. Cell transfection was performed using TurboFect transfection reagent (Thermo Scientific) according to the manufacturer’s instruction. The transfection efficiency was detected after 48 h post-transfection. The lentivirus plasmid pLL3.7-p110α was transfected to obtain the sh-p110α stable cell line. The primers’ sequences of sh-p110α were as follows: p110α-sh1: 5′-TTTAACAGAGCAGATTGAAAG-3′, p110α-sh2: 5′-TATTTCCTGGCCTCTCTGAAC-3′. PLVX-IRES was inserted with p110α and PRMT1 to acquire the stable overexpression in HepG2 and Huh7 cells. All cells were selected with puromycin for 1 week to acquire positive cell lines. All cell lines were demonstrated to be Mycoplasma-negative by PCR assays. Cell lines were detected every three months. All cells used for experiments were maintained for less than 20 passages.

For the design of sgRNA in ZhangFeng library, sgRNA targeting PRMT1 5′-CCGGCAGTACAAAGACTACAA-3′, was inserted in CRISPR vector Cas9-puro-PX459. HepG2 and Huh7 cells were transfected and were selected with puromycin. Then, transfected cells were cultured for 2 weeks and detected the efficiency of knockout expression.

### Western blotting and antibodies

Cells were lysed in RIPA lysis buffer (Thermo Scientific) including protease inhibitor cocktail (Sigma), and protein concentrations were measured using the BCA protein assay kit (Pierce). A total of 25 to 60 μg of protein was separated by SDS-PAGE and were transferred to nitrocellulose membranes (Pall). The primary antibodies used were as follows: anti- p110α (Santa Cruz, sc-293172), anti-p85α (Santa Cruz, sc-1637), anti-G3BP1 (Santa Cruz, sc-365338), anti-RPS6 (Santa Cruz, sc-74459), anti-RPL4 (Santa Cruz, sc-100838), anti-PABP (Santa Cruz, sc-166381), anti-eIF2α (Santa Cruz, sc-133132), anti-p-eIF2α (ABclonal, AP0745) and anti-β-actin (ABclonal, AC026). The secondary antibodies used were as follows: anti-rabbit IgG DyLight 800-conjugated (Rockland, 611-145-002) and anti-mouse IgG antibody DyLight 800-conjugated (Rockland, 610-145-121). The Odyssey infrared imaging system (LI-COR Bioscience, Lincoln, NE) was subjected to acquire the infrared fluorescence image.

### Chemical reagents, SG induction, and quantification

Chemical reagents performed for SG induction included sodium arsenite (AS, Sigma-Aldrich, S7400), H_2_O_2_ (TGREAG, 106057), sorbitol (AMRESCO, 0691), and NaCl (HARVEYBIO, SR4299). SGs were induced in cells by treatment with AS (500 μM for 1 h, H_2_O_2_ (1 mM for 1 h), NaCl (0.2 M for 30 min), or sorbitol (0.4 M for 30 min). SGs in cells were scored by manual counting utilizing fluorescence microscopy with G3BP1 and eIF4G as SG markers, and only cells with granules co-stained for these markers were identified as SGs, and a minimum of three granules per cell was considered for a positive score. In the cell experiments with Flag-tagged p110α deletants or mutants, only Flag-positive cells were considered, and the number of granules in an individual cell was counted.

### Immunoprecipitation

Cells were harvested and rinsed with ice-cold PBS. Cells were then lysed in IP lysis buffer (25 mM Tris-HCl (pH 7.4), 1% Nonidet P-40, 150 mM NaCl, 1 mM EDTA, 5% glycerol and 1% protease inhibitor cocktail). In some special experiments, IP lysis buffer contained 40 μg/ml RNase A with or without EE buffer (2 mM EDTA, 2.5 mM EGTA), and with or without 5 mM MgCl2. All proteins were incubated with antibodies and protein A-Sepharose (GE Healthcare) and were rotated at 4 °C overnight. The beads were washed with IP lysis buffer three times and denatured by SDS loading buffer and boiling for 10 min at 95 °C. Western blotting was performed to detect precipitated proteins.

### Immunofluorescence assay

The cells were grown and stressed as indicated in 3.5 cm confocal dishes and washed with PBS. Then, cells were fixed using 4% paraformaldehyde for 15 min and permeabilized in 0.1% Triton X-100/PBS for 15 min under room temperature. After washing with PBS for three times, cells were blocked by 1% bovine serum albumin prepared with PBS for 1 h under room temperature and then incubated with primary antibodies overnight at 4 °C. The next day, cells were incubated with the secondary antibodies labeled with Alexa Flour 549 (anti-mouse IgG) and Alexa-Flour 488 (anti-rabbit IgG). The nuclei were stained with 4,6-diamidino-2-phenylindole (DAPI) at final concentration of 1 μg/ml for 1 min. Images were photographed using a ZEISS fluorescence microscope. The primary antibodies used for immunofluorescence analyses are as follows: anti-G3BP1 (ABclonal, A3968) and anti-eIF4G (ABclonal, A7552).

### GST pull-down

Control GST and GST-labeled proteins were expressed in Escherichia coli strain BL21 (DE3) and purified by sonication, followed incubating with glutathione-Sepharose beads (GE Healthcare) overnight at 4 °C with rocking. The next day, His-tagged proteins were added, and the incubation continued for an additional for 4 h at 4 °C. After incubation, beads were washed with ice cold elution buffer, boiled in SDS-PAGE loading buffer and finally analyzed by western blotting with the indicated antibodies.

### Cell apoptosis

Cell apoptosis was conducted using the Annexin V-FITC/PI (propidium iodide) Apoptosis Detection Kit (KeyGEN BioTECH, KGA108). Briefly, exponentially growing cells were digested using trypsin without EDTA and washed with ice PBS. Cells were then resuspended in 500 μL of binding buffer and double-stained with 5 μL Annexin V-FITC and 5 μL PI at room temperature in the dark for 15 min. Finally, a flow cytometer (BD Biosciences) was applied to analyze apoptotic cells.

### In vitro methylation

Briefly, the purified His-PRMT1 and GST—p110α were incubated together with Histone methyltransferase (HMT) buffer (25 mM NaCl, 25 mM Tris-HCl pH 8.8, 2 μM SAM). The mixtures were gently agitated at 37 °C for 2 h and then subjected to SDS-PAGE and immunoblot analysis using the anti-ADMA antibody.

### Preparation of PEI-R612F and MPN-R612F

Added approximately 2 μg of R612F plasmid to 3.97 μg of PEI (25 kDa, 1 μg/μl) (at N/P ratio of 15), then diluted this mixture to 100 μl with 10 mM PBS. The solution was vortexed for about 10 s and incubated for 30 min at 37 °C to generate the PEI-R612F complex. Diluted the mixture to 1 mL using complete culture medium before adding cells and used immediately after preparation. For preparation of MPN-R612F, added 5 μl of FeCl_3_ (4.8 mM) to the prepared PEI-R612F complexes and vortexed for 5 s. Then, 5 μl of tannic acid (4.8 mM) was added and vortexed for 5 s. Finally, 20 μl Tris buffer (pH = 8.0) was added to the mixture and vortexed for 5 s to form MPN-R612F nanocomplexes. Similarly, the complex was diluted to 1 mL with complete medium before applying to cells.

### Cell viability

Approximately 5 × 10^3^ HepG2 cells were seeded in 96-well plates. MPN-R612F (with a R612F content of 2 μg), PEI-R612F (with a R612F content of 2 μg), sorafenib (10 μM) or sorafenib combined with MPN-R612F were added into cells. After treatment for 24 h, the culture medium was discarded and replaced with 200 μl of fresh culture medium and 15 μl of MTT solution (5 mg/mL in PBS buffer) with coincubation for 4 h. After that, the medium was replaced with 200 μL of DMSO, and the cell viability was measured using a microplate reader at 490 nm.

### Fluorescein-labeling of plasmids and lysosomal escape detection

CMV2-flag-G418-R612F plasmid was labeled by fluorescein probe utilizing the Label-IT Nucleic Acid Labeling Kits (Mirus Bio, USA, MIR3225) in accordance with the manufacturer’s protocol. Briefly, nucleic acid plasmid (1 mg/mL) and Label-IT reagents (100 μL/mL) were mixed in the buffer and then incubated the reaction at 37 °C for 1 h. Purified samples using G50 microspin purification columns and stored at −20 °C protected from light.

Fluorescein-labelled R612F was incubated with MPN or PEI to generate MPN-R612F or PEI-R612F and added into cells for 1, 2, 4, and 8 h before final treatment. Then, cells were collected and washed with cold PBS twice, following stained with lysotracker Red (Beyotime, Shanghai) at 37 °C for 1 h. Afterwards, we used Hoechst 33342 (Beyotime, Shanghai) to stain the cell nuclei. Images were observed through confocal laser scanning microscopy (TCS, SP8, Leica, Germany).

### Transmission electron microscope assay

In order to assess the shape and size of MPN-R612F and PEI-R612F, a scanning transmission electron microscope (TEM) detector was performed.

### In vivo anticancer therapy

To assess the role of MPN-R612F in tumorigenesis, five-week-old female Balb/c nude mice were obtained from the Health Science Center of Peking University. Mice are raised and maintained under a 12-h dark/light cycle, with free food and water provided. In detail, about 5 × 10^6^ HepG2 cells were suspended in 100 μL PBS and then were subcutaneously injected into virgin female Balb/c nude mice. When the tumor volume reached to 50 mm^3^, mice were randomly divided into five groups (5 mice per group). Each group of mice was injected with 100ul of relevant solutions through the tail vein at first, fifth, and tenth days, with the following solutions: PBS, MPN-vector, MPN-R612F, sorafenib, sorafenib + MPN-R512F. On the 30th day, all mice were sacrificed and tumors were harvested for further analysis. Animal experiments were performed according to Institutional Animal Care and Use Committee (IACUC) approved guidelines (LA2020230).

### TCGA and computational data analysis

To investigate the correlations between G3BP1 gene expression and p110α gene expression, we used RNA-Seq datasets GEPIA based on the UCSC Xena project (http://xena.ucsc.edu), which are computed by a standard pipeline. Pearson correlation test was used to analyze the correlation between G3BP1 and p110α in LIHC from TCGA patients.

### Statistical analysis

All experiments were performed at least three times and similar results were obtained. A two-tailed Student *t* test was performed to calculate the difference between two groups. Differences among multiple groups were calculated by the one-way ANOVA analysis. The data are expressed as mean ± SD (standard deviation). A value of at least *p* < 0.05 was considered statistically significant.

### Supplementary information

Supplemental Figure and Table Legends

Supplemental Figures

Supplemental Table

Original western blot figures

## Data Availability

All data of this paper are included in the Main Text and Supplementary Files.
