# Supplementary material for: Metal-polyphenol-network coated R612F nanoparticles reduce drug resistance in hepatocellular carcinoma by inhibiting stress granules
Source: Cell Death Discov. 2024 Aug 28;10:384. doi: 10.1038/s41420-024-02161-6 (PMC11358291; doi:10.1038/s41420-024-02161-6)
Supplement: Supplementary file 1 — Supplemental Figure and Table Legends [file 41420_2024_2161_MOESM1_ESM.docx]

**Supplemental Figure Legends:**

**Supplemental Fig 1**. **The associations of subunits of PI3K**. The correlation analysis of G3BP1 and p110β, p110δ, p85α or p85β from TCGA public database. Pearson test was performed. LIHC, Liver Hepatocellular Carcinoma.

**Supplemental Fig 2. p110α regulates SGs formation after stress recovery.** A. SGs visualization under stress recovery after p110α overexpression. HepG2 and Huh7 cells were transfected with Flag- p110α, and stained for G3BP1 (red)/eIF4G (green) as SG markers. B. SGs visualization under stress recovery after p110α knockdown. HepG2 and Huh7 cells were stably transfected with pLL3.7-p110α through lentiviral infection, and stained for G3BP1 (red)/eIF4G (green) as SG markers. Bars denote the average percentage of cells with more than three SGs. Data represent the mean ± SD. n = 3. Scale bars = 10 μm. **p* < 0.05.

**Supplemental Fig 3. The nanoparticle MPN-R612F was designed to kill cancer cells combined with sorafenib.** A. The quantification of Fig 7A. B. The dissociation of MPN-R612F by EDTA, urea, NaCl, and Tween 20. C. Double-immunofluorescence analysis of SGs formation in HepG2 cells treatment with PEI-R612F or MPN-R612F, then administrating with AS (500 μM, 1 h), and stained with G3BP1 (red)/p110α (green) as SG markers. Scale bar = 10 μm. D. Tumor volume of indicated groups. E. The quantification of Fig 7L. F. The effect of MPN-R612F on the content of ALT and AST in vivo. G. The effect of MPN-R612F on the content of CREA and UREA in vivo. H. Images of liver and kidney in each group of mice. I. H&E staining of liver and kidney in each group of mice. Data represent the mean ± SD. n = 3. **p* < 0.05, ***p* < 0.01.

**Supplemental Table. Primers used in the experiments are listed.**
