# Supplementary figures and images for: Metal-polyphenol-network coated R612F nanoparticles reduce drug resistance in hepatocellular carcinoma by inhibiting stress granules
Source: Cell Death Discov. 2024 Aug 28;10:384. doi: 10.1038/s41420-024-02161-6 (PMC11358291; doi:10.1038/s41420-024-02161-6)

# Supplemental Figure 1

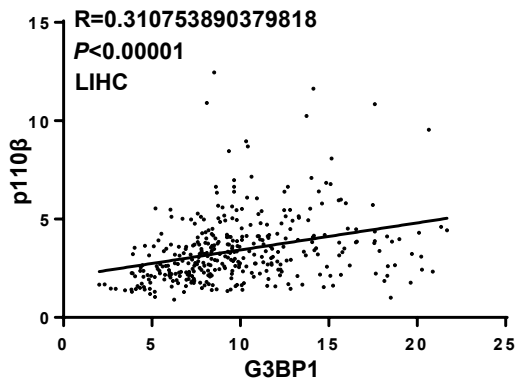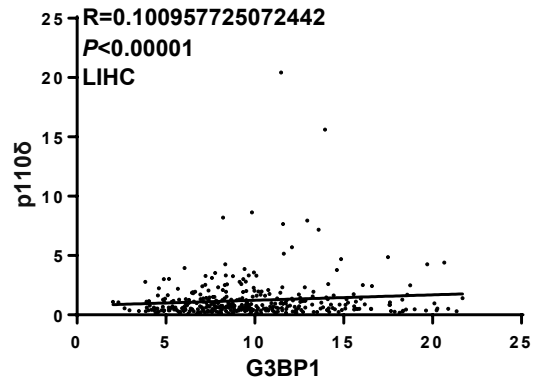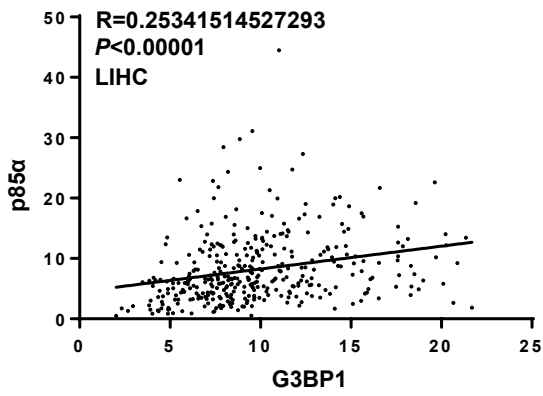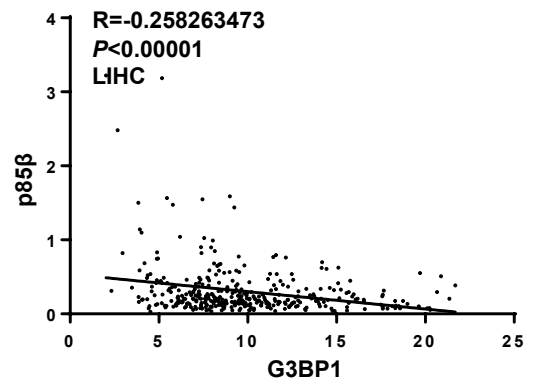

A

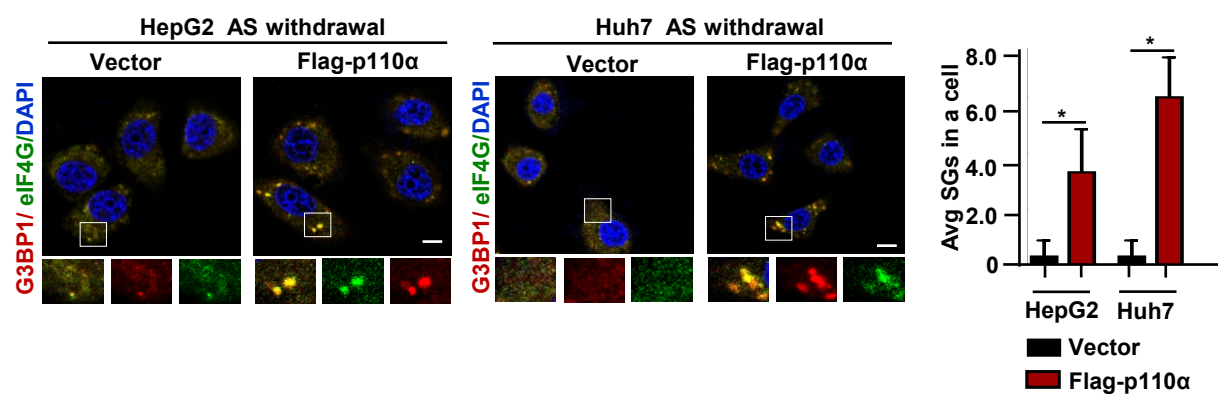

B

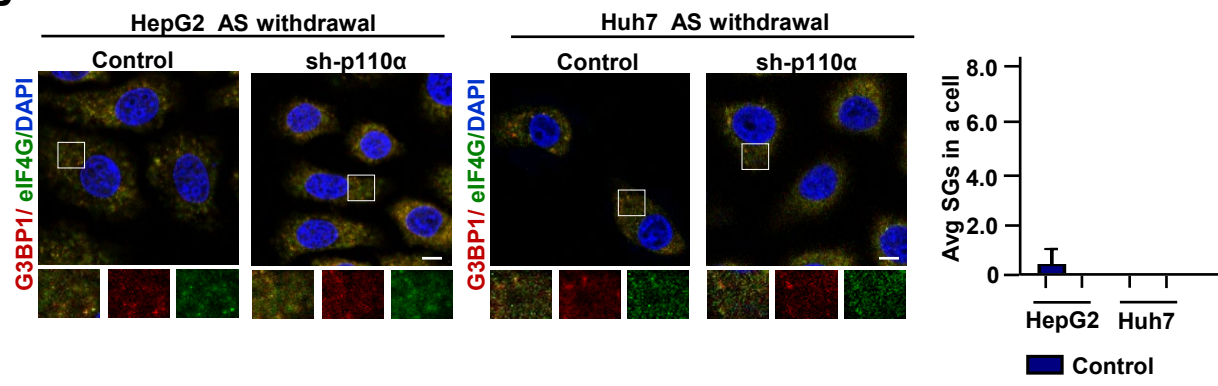

**A**

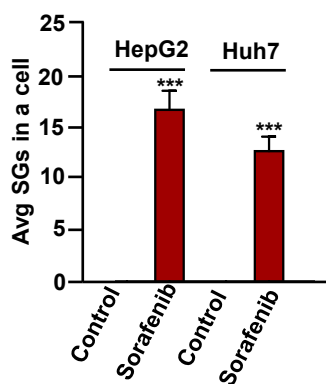

**B**

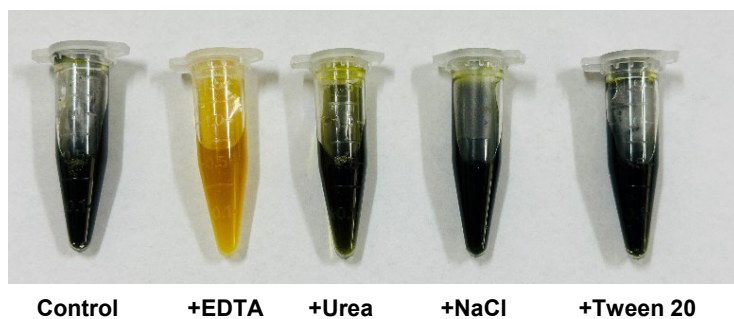

**C**

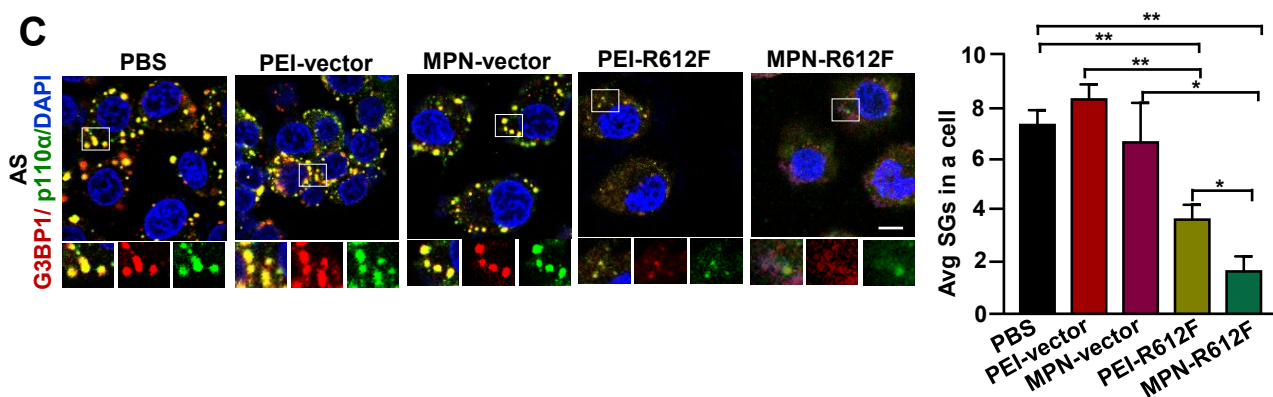

**D**

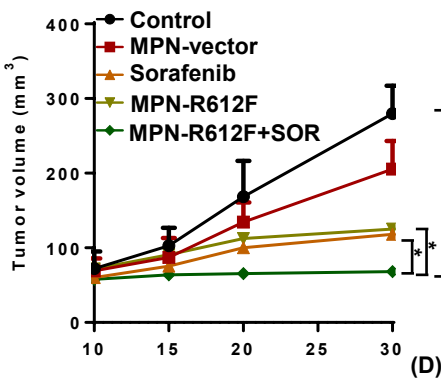

**E**

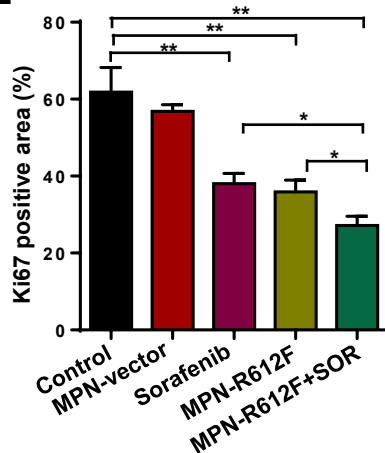

**F**

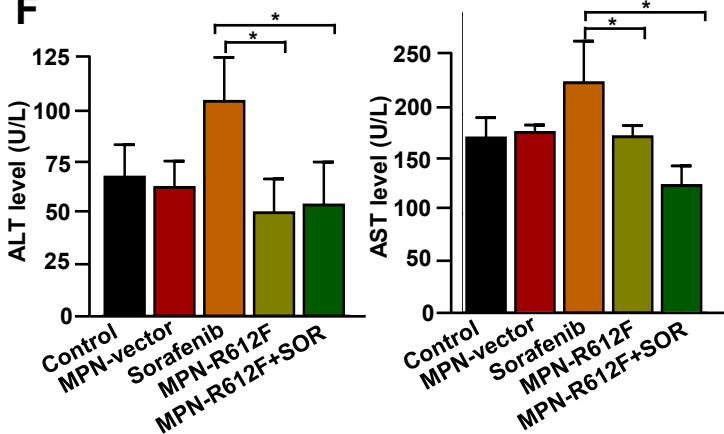

G

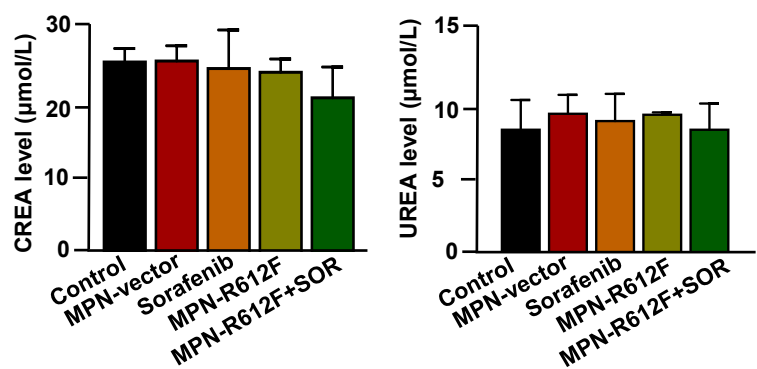

H

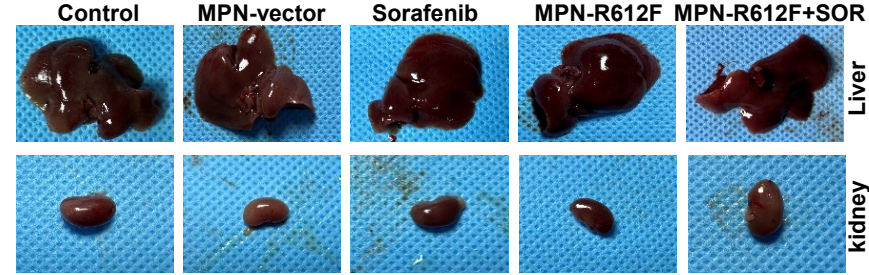

I

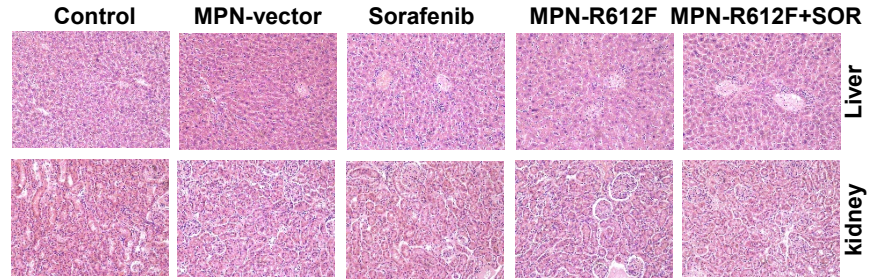

Supplement: Supplementary file 2 — Supplemental Figures [file 41420_2024_2161_MOESM2_ESM.pdf]
