# Supplementary material for: Metal-polyphenol-network coated R612F nanoparticles reduce drug resistance in hepatocellular carcinoma by inhibiting stress granules
Source: Cell Death Discov. 2024 Aug 28;10:384. doi: 10.1038/s41420-024-02161-6 (PMC11358291; doi:10.1038/s41420-024-02161-6)
Supplement: Supplementary file 3 — Supplemental Table [file 41420_2024_2161_MOESM3_ESM.docx]

**STable. Primers used in the experiments**

|  | **Forward Primer 5'-3'** | **Reverse Primer 5'-3'** |
| --- | --- | --- |
| **R612A** | **CCCAGATCCTATGGTTGCTGGTTTTGCTGT** | **AGCAACCATAGGATCTGGGTAATTACAGTCCAG** |
| **R612F** | **CCCAGATCCTATGGTTTTCGGTTTTGCTGT** | **GAAAACCATAGGATCTGGGTAATTACAGTCCAG** |
| **ΔABD** | **TGGGGCATCCACTTGGGCAACCGTGAAGAAAAG** | **TCAGTTCAATGCATGCTGTTTAATTGTGTGGAAGATCC** |
| **ΔRBD** | **ATATAATAAATTAGATAAAGAAAGCCTTTAT** | **TCAGTTCAATGCATGCTGTTTAATT** |
| **ΔC2** | **AAATCCCTTTGGGTTGATATGTCAGTGATT** | **TCAGTTCAATGCATGCTGTTTAATTGTGTG** |
| **ΔHelical** | **GGACTGAGTAACAGATGTGGGATGTATTTG** | **TCAGTTCAATGCATGCTGTTTAATTGTGTGGAAG** |
| **ΔKinase** | **ATGCCTCCACGACCATC ATCAG** | **ATCCATTTTTGTTGTGAGGTTTCCTAGTTG** |
