# Supplementary material for: Metal-polyphenol-network coated R612F nanoparticles reduce drug resistance in hepatocellular carcinoma by inhibiting stress granules
Source: Cell Death Discov. 2024 Aug 28;10:384. doi: 10.1038/s41420-024-02161-6 (PMC11358291; doi:10.1038/s41420-024-02161-6)
Supplement: Supplementary file 4 — Original western blot figures [file 41420_2024_2161_MOESM4_ESM.pdf]

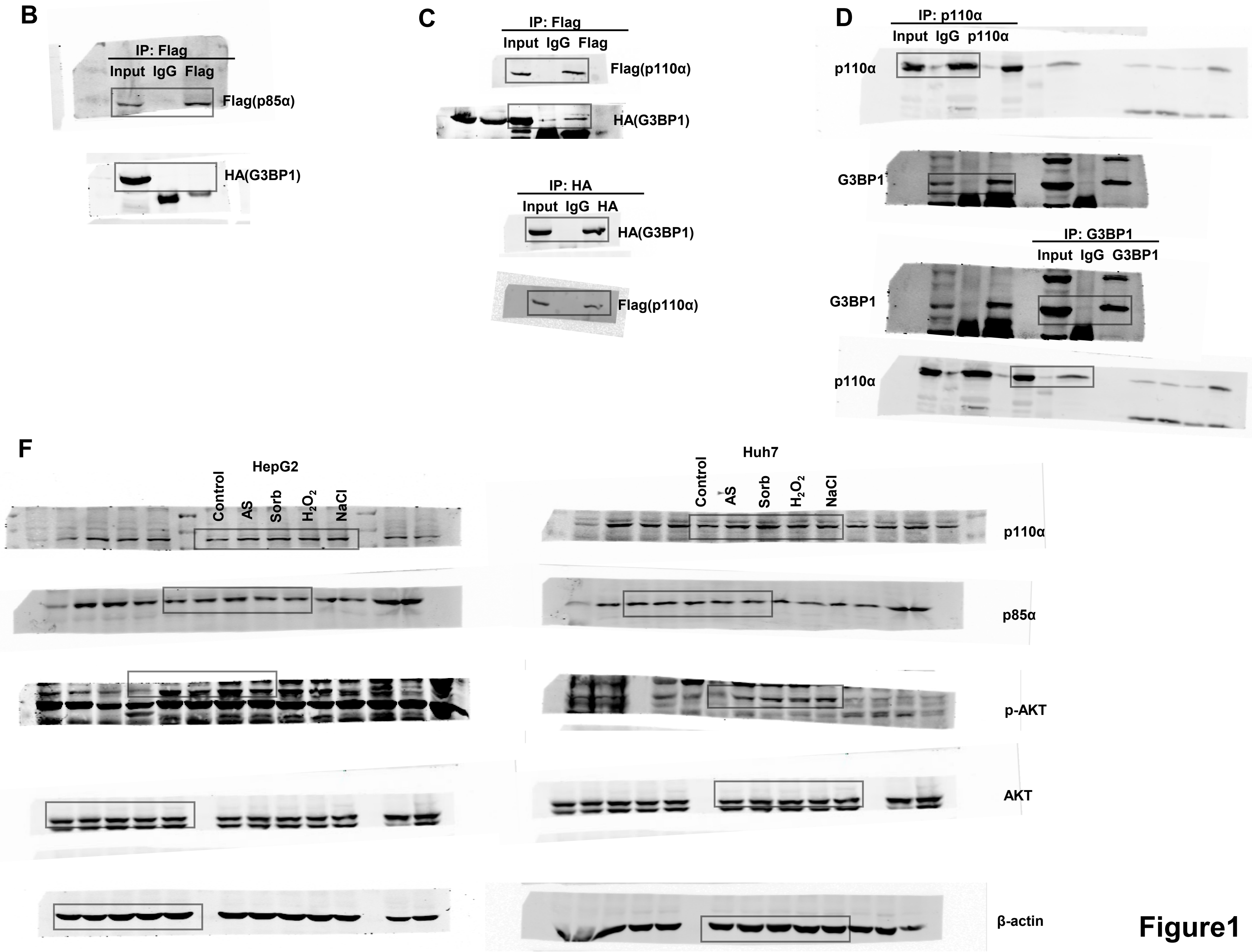
B
IP: Flag
Input IgG Flag
Flag(p85α)
HA(G3BP1)
C
IP: Flag
Input IgG Flag
Flag(p110α)
HA(G3BP1)
IP: HA
Input IgG HA
HA(G3BP1)
Flag(p110α)
D
IP: p110α
Input IgG p110α
p110α
G3BP1
IP: G3BP1
Input IgG G3BP1
G3BP1
p110α
F
HepG2
Control
AS
Sorb
H2O2
NaCl
Huh7
Control
AS
Sorb
H2O2
NaCl
p110α
p85α
p-AKT
AKT
β-actin

Figure1

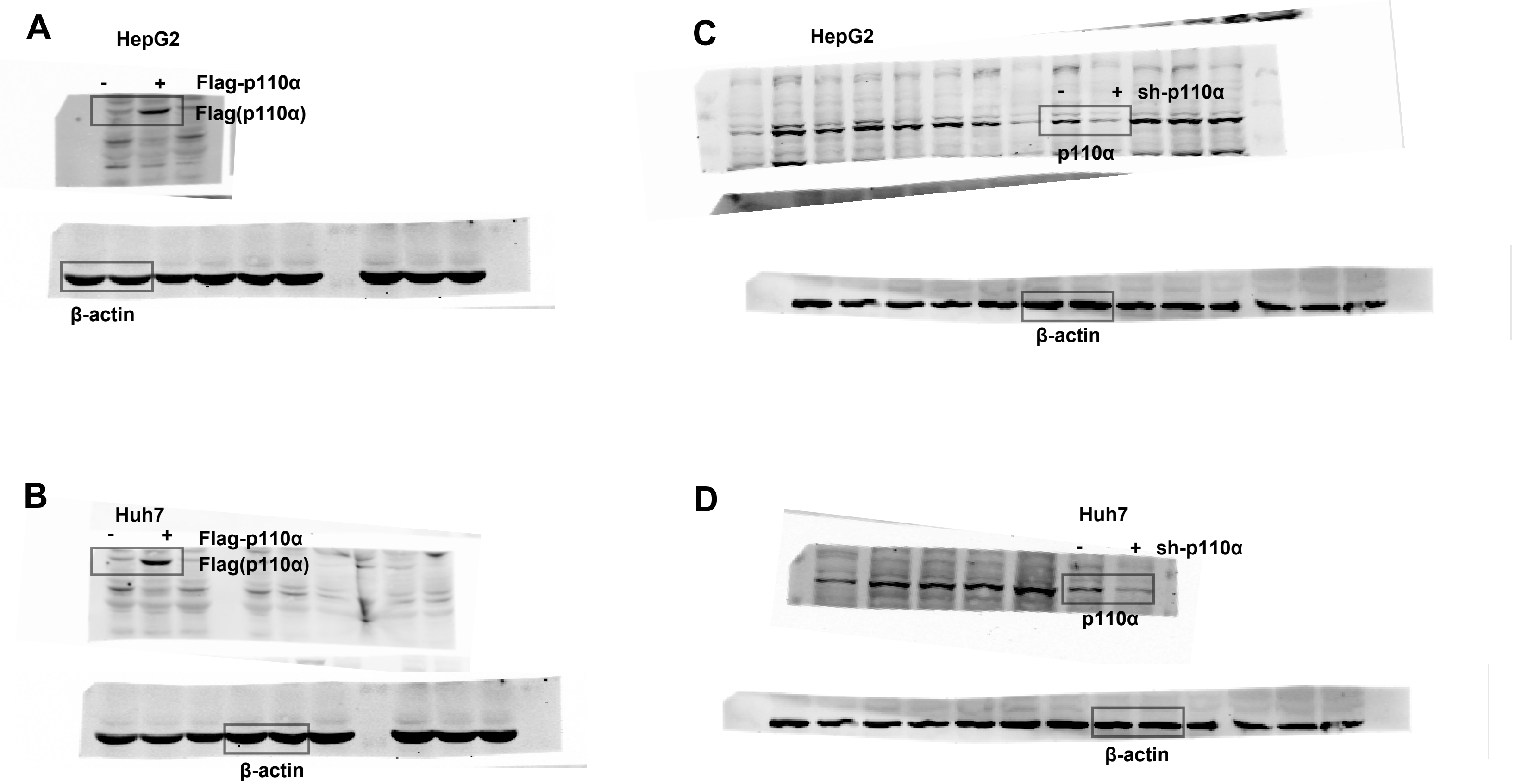
A
HepG2
- + Flag-p110α
Flag(p110α)
β-actin
C
HepG2
- + sh-p110α
p110α
β-actin
B
Huh7
- + Flag-p110α
Flag(p110α)
β-actin
D
Huh7
- + sh-p110α
p110α
β-actin

Figure2

A

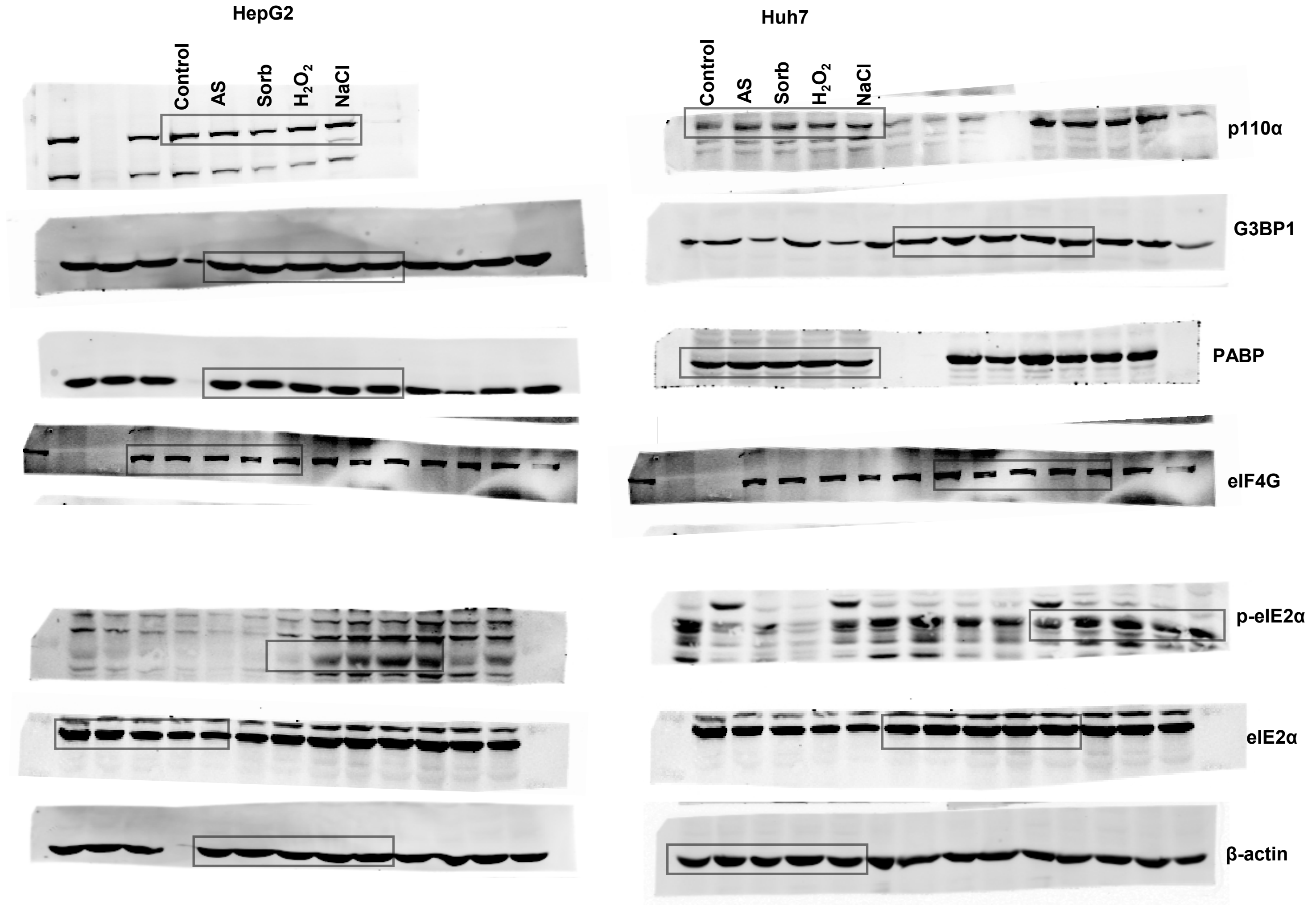
HepG2
Huh7
Control
AS
Sorb
H_2O_2
NaCl
p110α
G3BP1
PABP
eIF4G
p-eIE2α
eIE2α
β-actin

Figure3

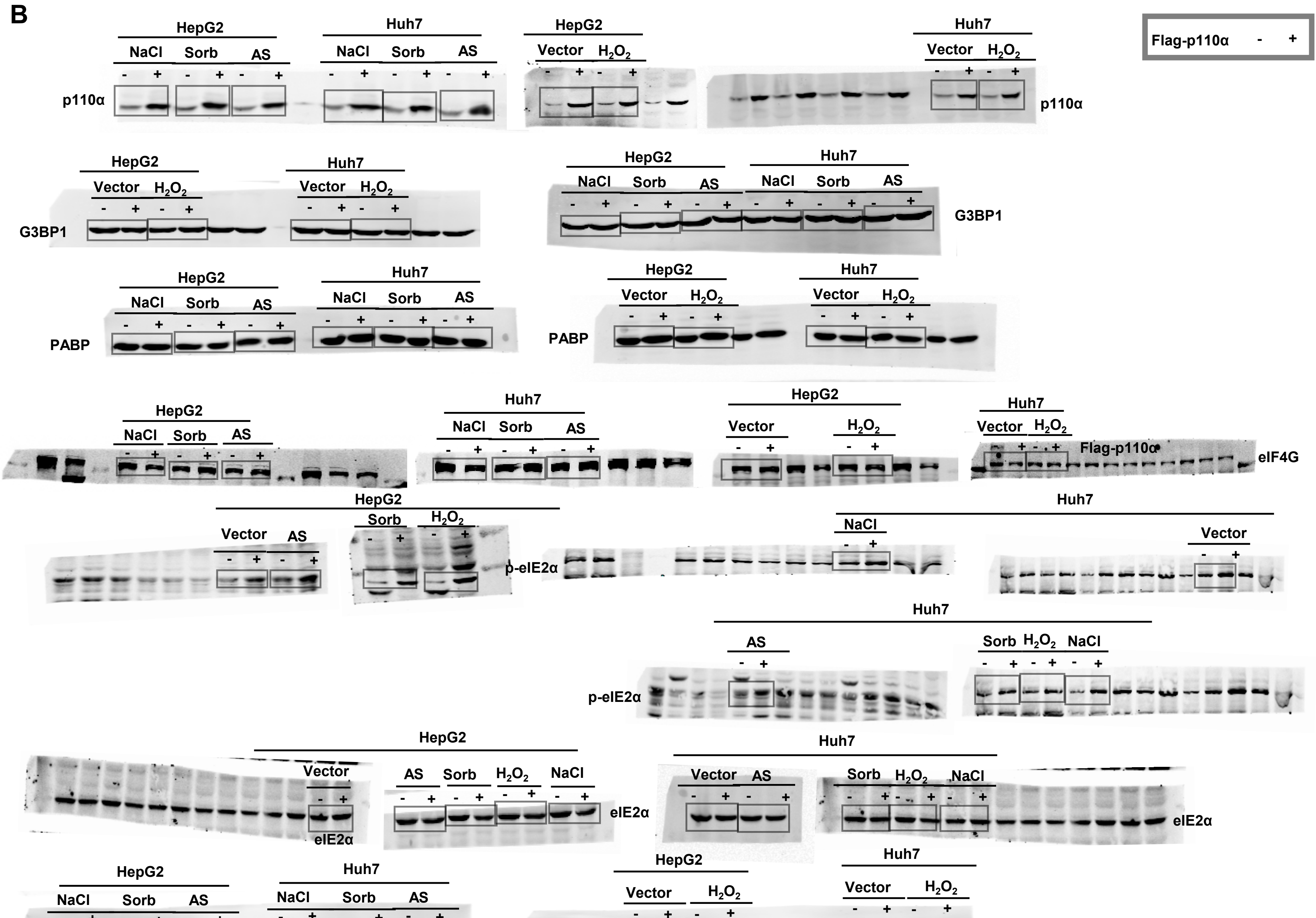
B
HepG2
Huh7
NaCl
Sorb
AS
Vector
H_2O_2
Flag-p110α
p110α
G3BP1
PABP
eIF4G
p-eIE2α
eIE2α
β-actin

Figure3

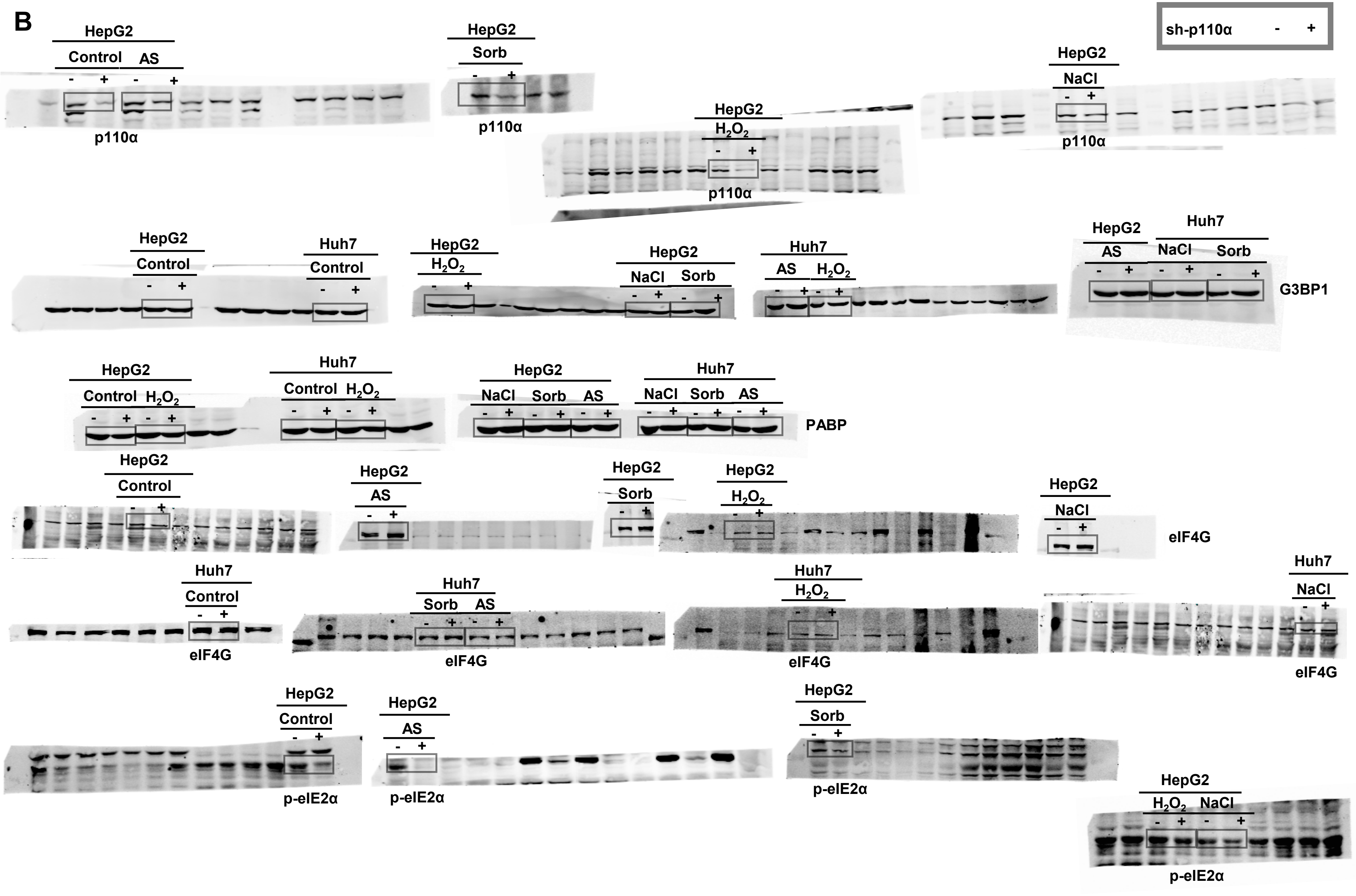
B
sh-p110α - +
HepG2
Control AS
p110α
Sorb
H2O2
NaCl
Huh7
G3BP1
PABP
eIF4G
p-eIE2α

Figure3

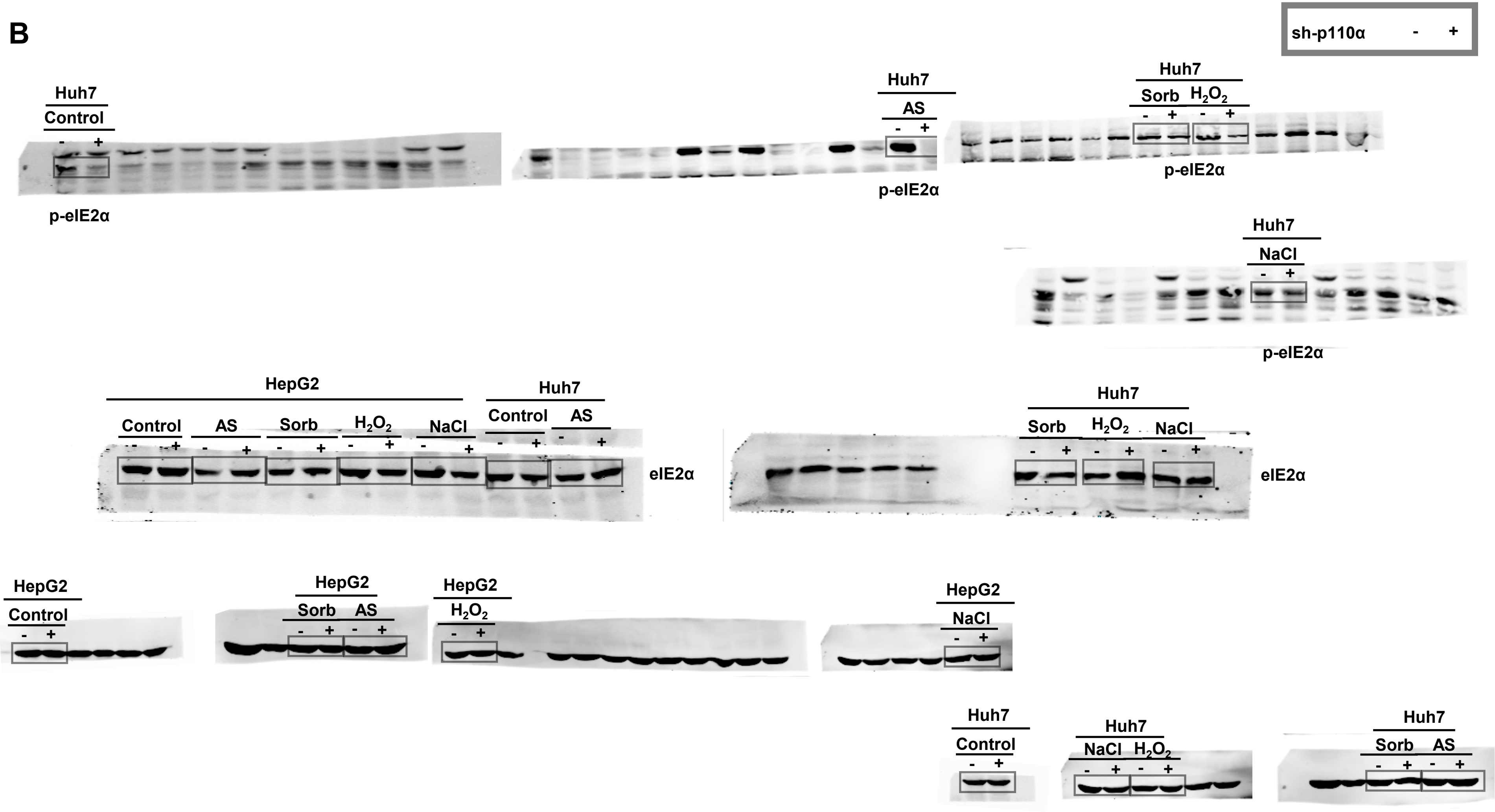
B
sh-p110α - +
Huh7
Control
- +
p-eIE2α
Huh7
AS
- +
p-eIE2α
Huh7
Sorb H$_2$O$_2$
- + - +
p-eIE2α
Huh7
NaCl
- +
p-eIE2α
HepG2
Control AS Sorb H$_2$O$_2$ NaCl
Huh7
Control AS
- + - + - + - + - + - + - +
eIE2α
Huh7
Sorb H$_2$O$_2$ NaCl
- + - + - +
eIE2α
HepG2
Control
- +
HepG2
Sorb AS
- + - +
HepG2
H$_2$O$_2$
- +
HepG2
NaCl
- +
Huh7
Control
- +
Huh7
NaCl H$_2$O$_2$
- + - +
Huh7
Sorb AS
- + - +

Figure3

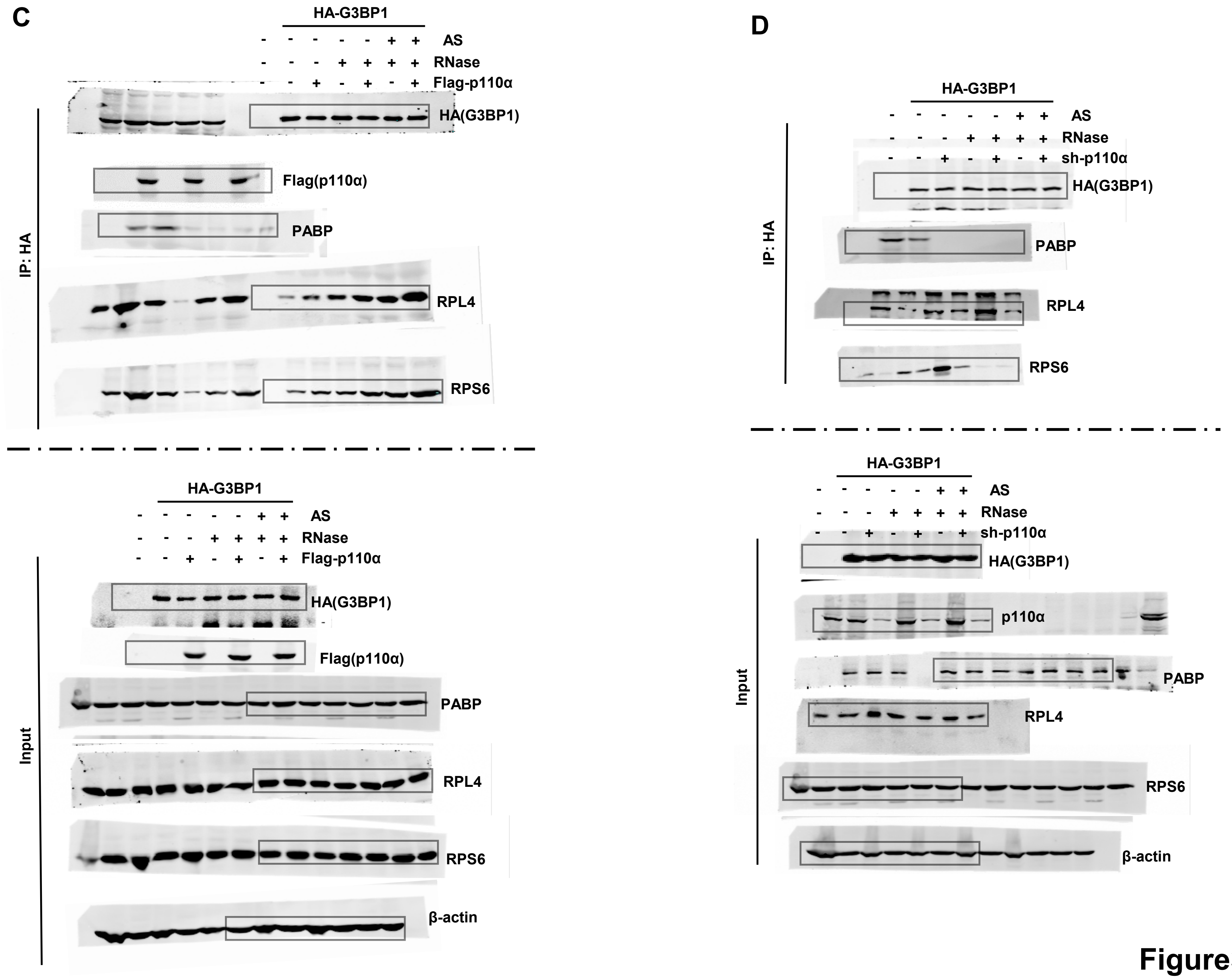
C
HA-G3BP1
AS
RNase
Flag-p110α
IP: HA
HA(G3BP1)
Flag(p110α)
PABP
RPL4
RPS6
Input
HA(G3BP1)
Flag(p110α)
PABP
RPL4
RPS6
β-actin
D
HA-G3BP1
AS
RNase
sh-p110α
IP: HA
HA(G3BP1)
PABP
RPL4
RPS6
Input
HA(G3BP1)
p110α
PABP
RPL4
RPS6
β-actin

Figure3

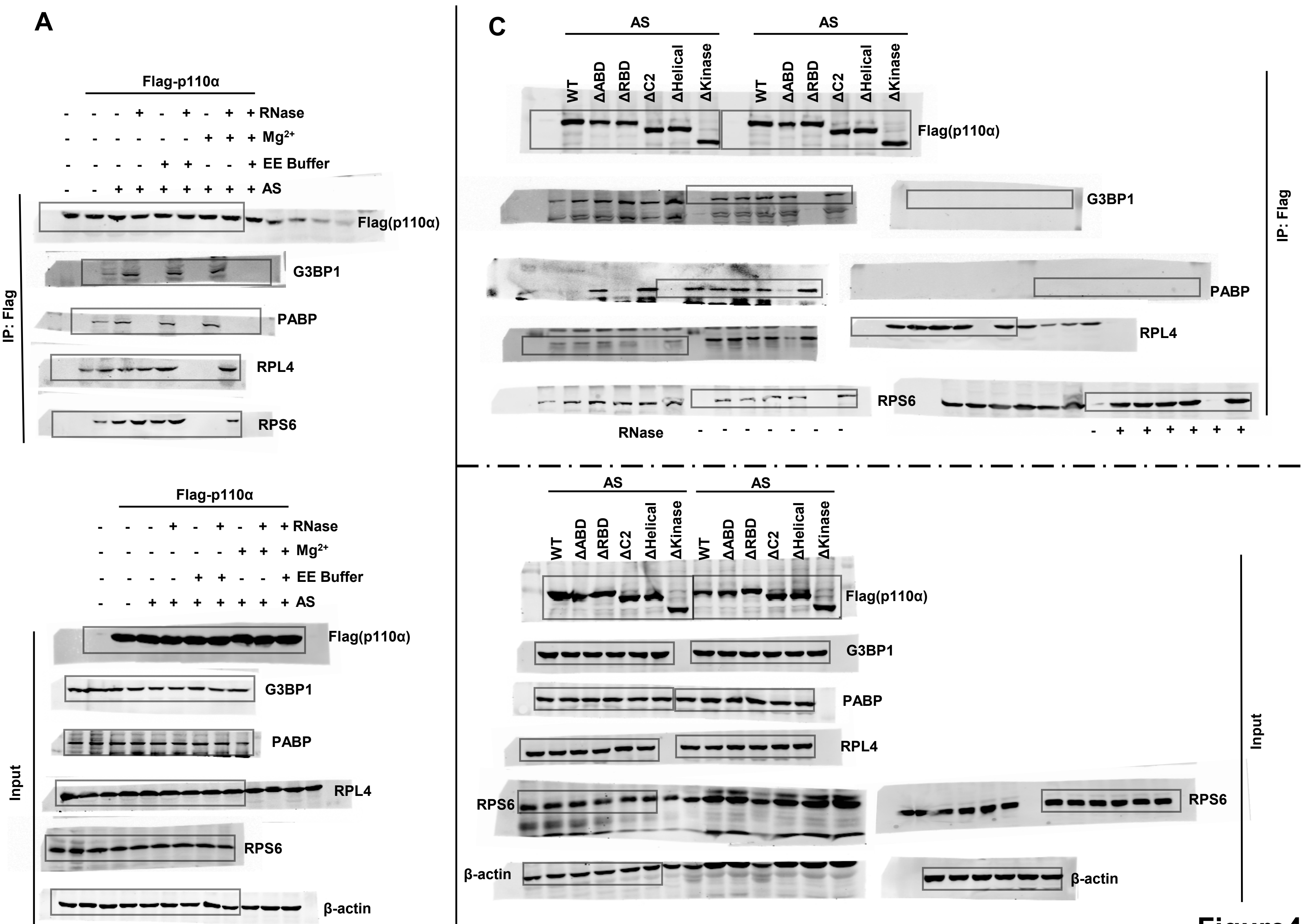
A
Flag-p110α
RNase
Mg2+
EE Buffer
AS
Flag(p110α)
G3BP1
PABP
RPL4
RPS6
IP: Flag
Input
β-actin
C
AS
WT
ΔABD
ΔRBD
ΔC2
ΔHelical
ΔKinase
RNase

Figure4

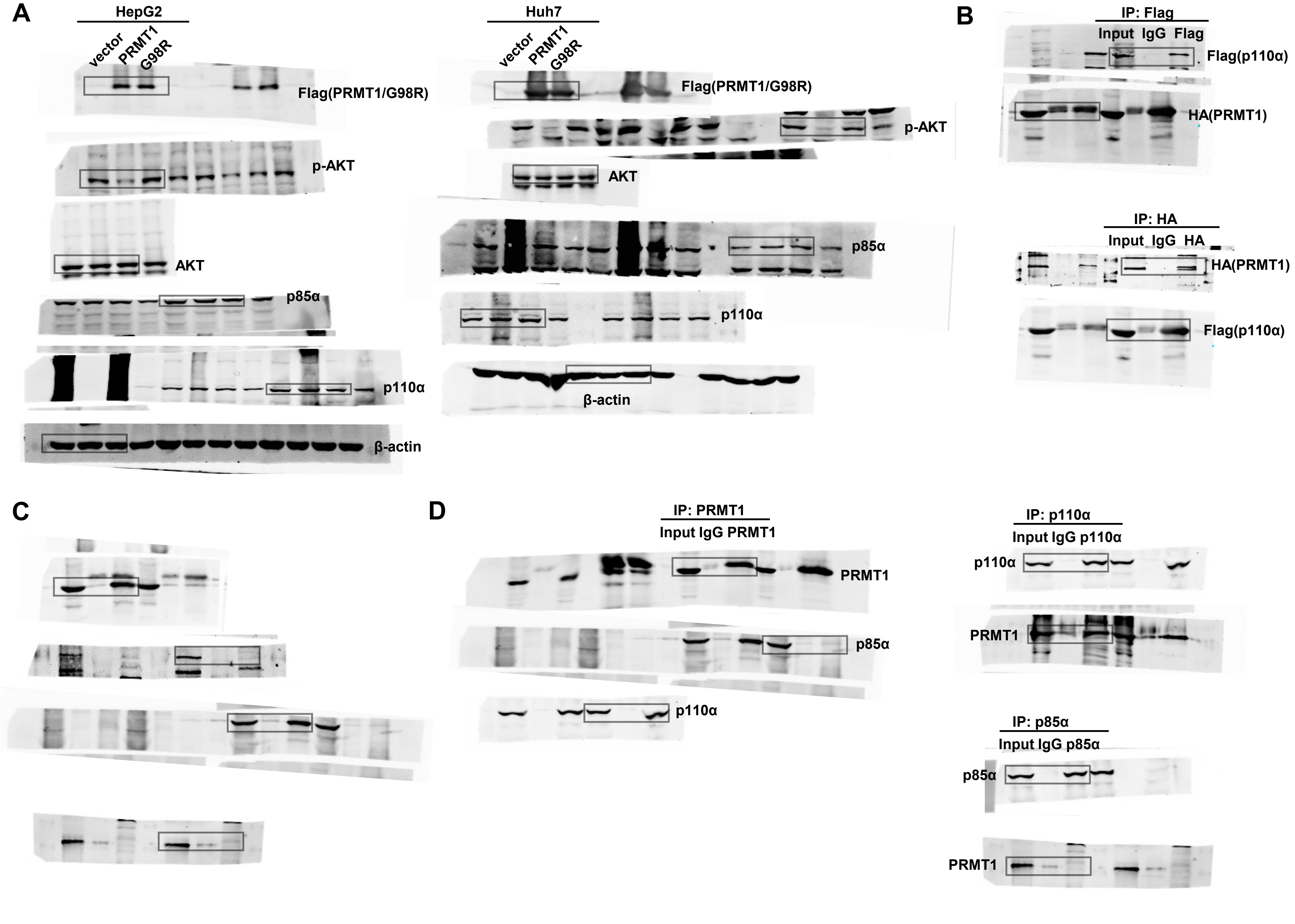
A
HepG2
vector
PRMT1
G98R
Flag(PRMT1/G98R)
p-AKT
AKT
p85α
p110α
β-actin
Huh7
vector
PRMT1
G98R
Flag(PRMT1/G98R)
p-AKT
AKT
p85α
p110α
β-actin
B
IP: Flag
Input IgG Flag
Flag(p110α)
HA(PRMT1)
IP: HA
Input IgG HA
HA(PRMT1)
Flag(p110α)
C
D
IP: PRMT1
Input IgG PRMT1
PRMT1
p85α
p110α
IP: p110α
Input IgG p110α
p110α
PRMT1
IP: p85α
Input IgG p85α
p85α
PRMT1

Figure5

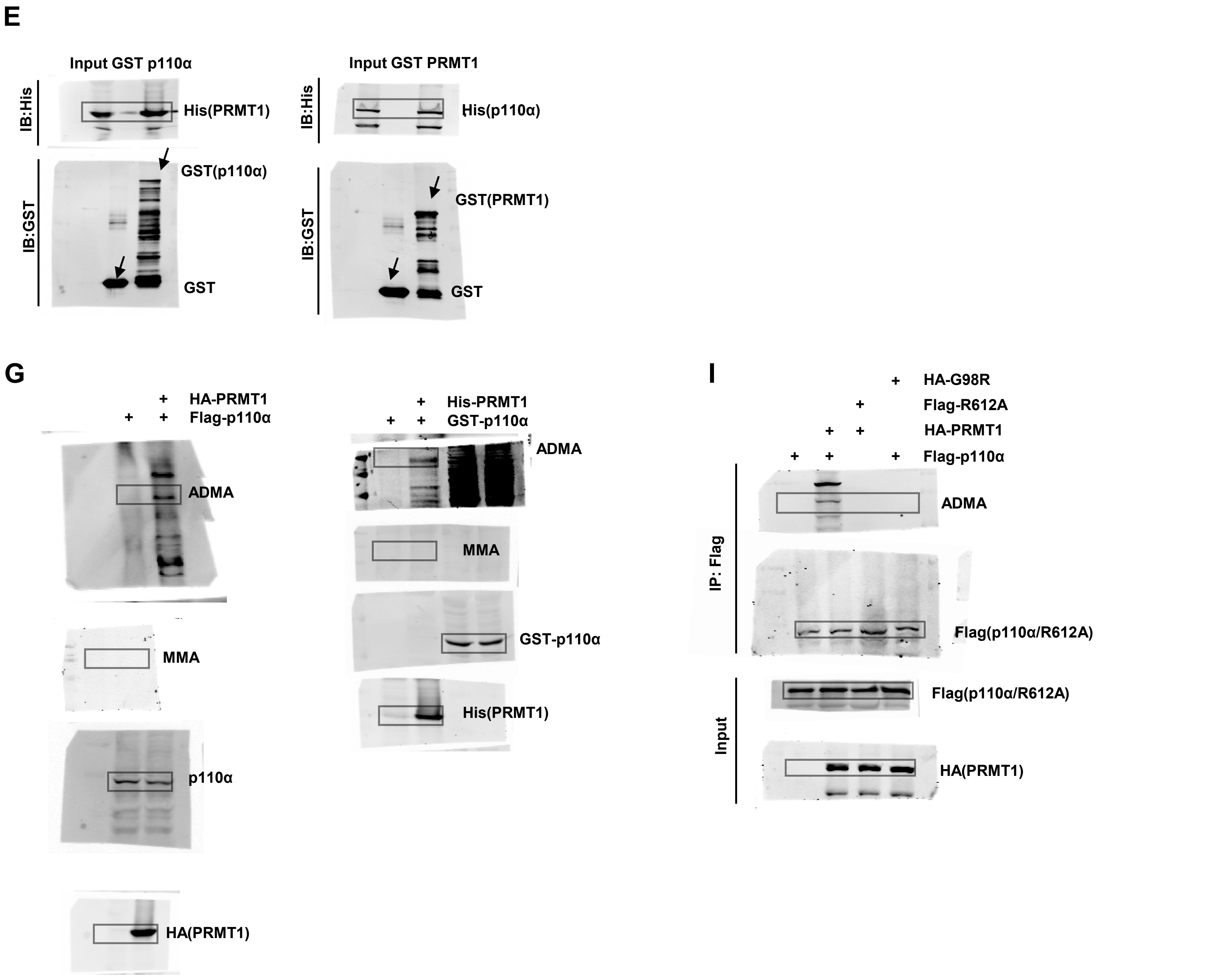
E
Input GST p110α
Input GST PRMT1
IB:His
His(PRMT1)
His(p110α)
IB:GST
GST(p110α)
GST(PRMT1)
GST
G
HA-PRMT1
Flag-p110α
ADMA
MMA
p110α
HA(PRMT1)
His-PRMT1
GST-p110α
His(PRMT1)
I
HA-G98R
Flag-R612A
IP: Flag
Flag(p110α/R612A)
Input
HA(PRMT1)

Figure5

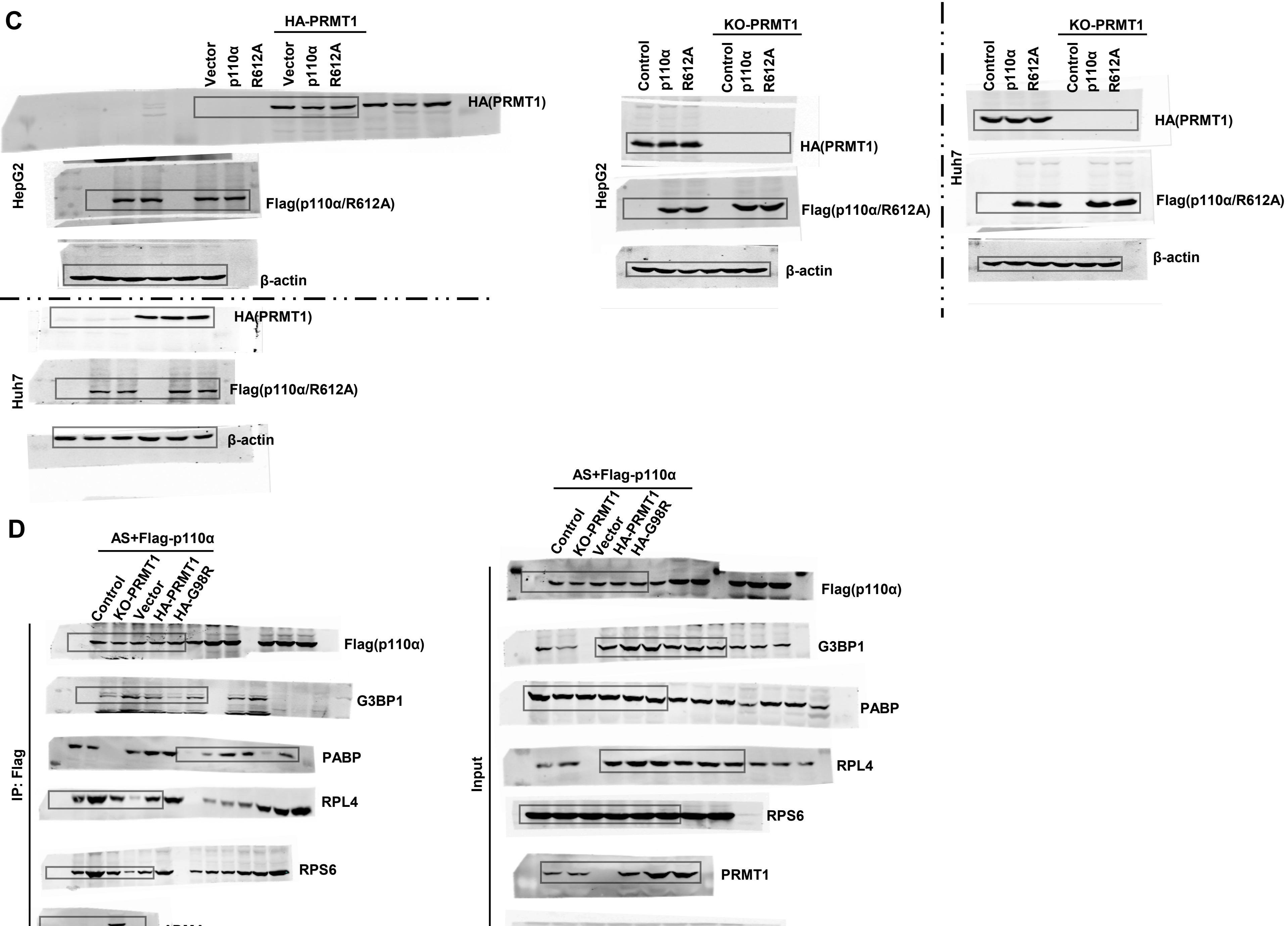
C
HA-PRMT1
Vector
p110α
R612A
Vector
p110α
R612A
HA(PRMT1)
HepG2
Flag(p110α/R612A)
β-actin
HA(PRMT1)
Huh7
Flag(p110α/R612A)
β-actin
KO-PRMT1
Control
p110α
R612A
Control
p110α
R612A
HA(PRMT1)
HepG2
Flag(p110α/R612A)
β-actin
KO-PRMT1
Control
p110α
R612A
Control
p110α
R612A
HA(PRMT1)
Huh7
Flag(p110α/R612A)
β-actin
D
AS+Flag-p110α
Control
KO-PRMT1
Vector
HA-PRMT1
HA-G98R
IP: Flag
Flag(p110α)
G3BP1
PABP
RPL4
RPS6
ADMA
AS+Flag-p110α
Control
KO-PRMT1
Vector
HA-PRMT1
HA-G98R
Input
Flag(p110α)
G3BP1
PABP
RPL4
RPS6
PRMT1
β-actin

Figure6

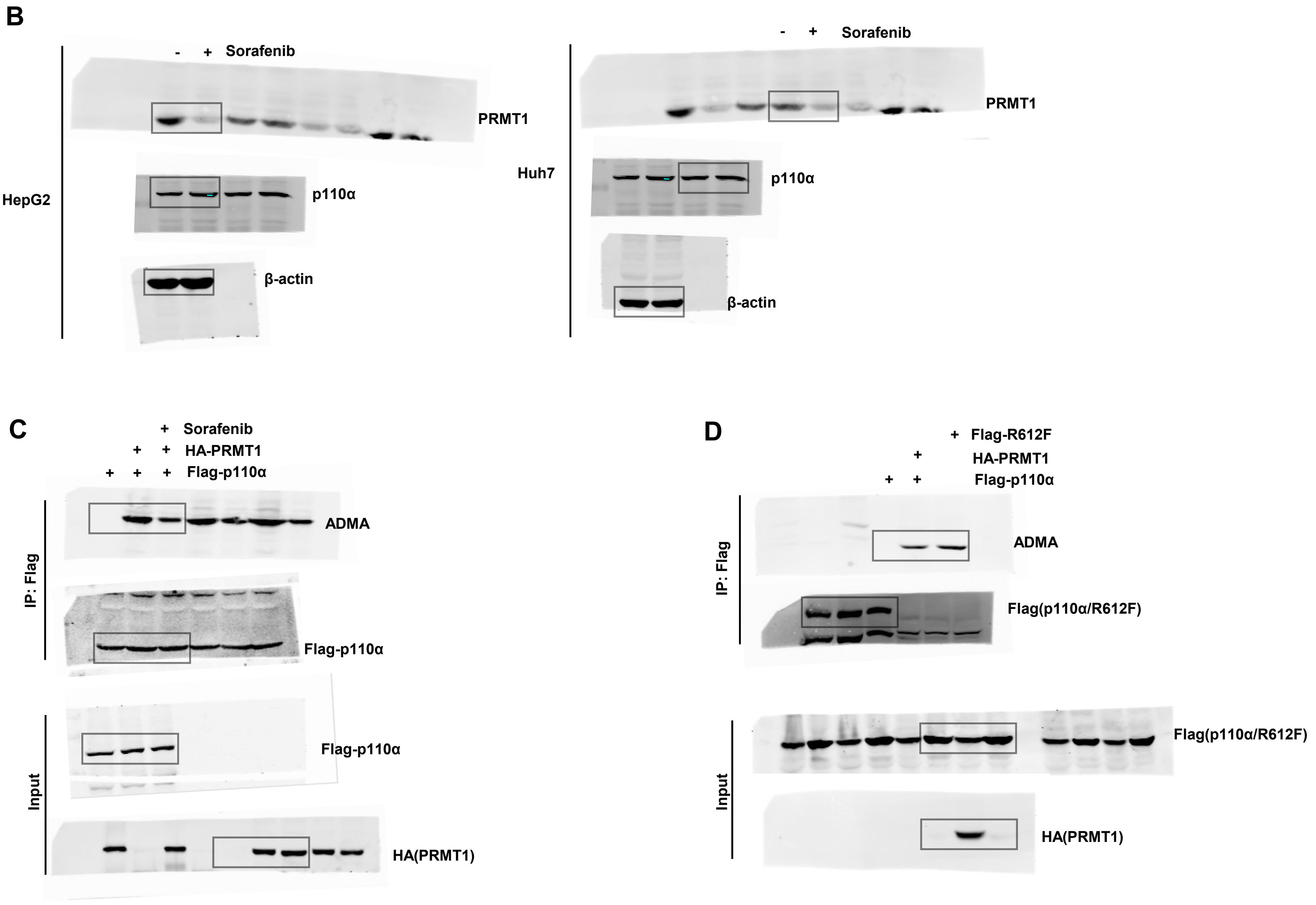
B
- + Sorafenib
PRMT1
p110α
β-actin
HepG2
- + Sorafenib
PRMT1
Huh7
p110α
β-actin
C
+ Sorafenib
+ + HA-PRMT1
+ + + Flag-p110α
ADMA
IP: Flag
Flag-p110α
Flag-p110α
Input
HA(PRMT1)
D
+ Flag-R612F
+ HA-PRMT1
+ + Flag-p110α
ADMA
IP: Flag
Flag(p110α/R612F)
Flag(p110α/R612F)
Input
HA(PRMT1)

Figure7
